# Multiple-component interventions to increase rotavirus vaccine uptake in children: a randomised controlled trial

**DOI:** 10.1016/j.lanwpc.2024.101153

**Published:** 2024-08-05

**Authors:** Karene Hoi Ting Yeung, Christy Ching Wun Yeung, Wing Hung Tam, King Shun Liu, Genevieve Po Gee Fung, E. Anthony S. Nelson

**Affiliations:** a School of Medicine, The Chinese University of Hong Kong, Shenzhen, Guangdong, 518172, PR China; b Department of Obstetrics and Gynaecology, Faculty of Medicine, The Chinese University of Hong Kong, Hong Kong, PR China; c Department of Paediatrics and Adolescent Medicine, United Christian Hospital, Hong Kong, PR China; d Department of Paediatrics, Faculty of Medicine, The Chinese University of Hong Kong, Hong Kong, PR China

**Keywords:** Rotavirus vaccine, Randomised controlled trial, Intervention, Hong Kong, Paediatrics, Equity

## Abstract

**Background:**

Rotavirus vaccine has not been included in the Hong Kong Government's Childhood Immunisation Programme. This randomised controlled trial examined whether a simple intervention package can increase rotavirus vaccine uptake in Hong Kong children.

**Methods:**

Postpartum mothers were recruited from two public hospitals in Hong Kong and randomly allocated into three groups using block randomisation, with block sizes kept unknown to investigators and research staff. Control-subjects received public rotavirus information. Subjects in intervention group 1 additionally received: key rotavirus information with a hyperlink to a webpage showing private clinics providing rotavirus vaccines and guidance on searching the clinics, and vaccination reminders. Subjects in intervention group 2 received the same intervention as group 1, plus tokens to receive free rotavirus vaccines at specific health centres. Rotavirus vaccination status was collected when children were approximately 8 months old. Maternal attitudes towards rotavirus vaccine were assessed at enrolment and at the end of the study. This trial has been registered in the Chinese Clinical Trial Register (Ref.:ChiCTR2000039791).

**Findings:**

From 16 February to 30 July 2021, 788 eligible mothers were recruited and randomly allocated to control group (n = 263), intervention group 1 (n = 263), and intervention group 2 (n = 262). The full intervention package (intervention group 2 relative to control group) increased rotavirus vaccine uptake by 1.7 times (95% confidence interval [CI] = 1.49–1.97) or by 33 percent-points (from 48% to 81% uptake). Provision of key rotavirus information with vaccination reminders (intervention group 1 relative to control group) and removal of financial barrier (intervention group 2 relative to intervention group 1) increased uptake by 1.17 times (95% CI = 0.99–1.38) or 8 percent-points, and by 1.46 times (95% CI = 1.29–1.66) or 25 percent-points, respectively.

**Interpretation:**

A multiple-component intervention package, and in particular providing free vaccine, could increase the uptake of rotavirus vaccine in Hong Kong children. The impact of the intervention package was greatest in low-income families, emphasising the importance of removing financial barriers to vaccination to promote equity. Incorporating rotavirus vaccine into the routine CIP could further protect more young children from rotavirus infection and improve equity.

**Funding:**

This work was supported by the Health and 10.13039/100012335Medical Research Fund by the 10.13039/100022720Health Bureau, Government of Hong Kong SAR [Ref.: 19180202].

**Research in context**

**Evidence before this study**

The uptake of the safe, effective and cost-saving rotavirus vaccine is low in Hong Kong. A local knowledge, attitudes and practices study in 2014 showed that higher income families were more likely to vaccinate their children with rotavirus vaccines. To the best of our knowledge, there have been no intervention studies or randomised controlled trials targeted to increase uptake of rotavirus vaccine alone in children, but some studies evaluated interventions to increase uptakes of all childhood vaccines included in national immunisation programmes. A review on interventions to increase paediatric vaccine uptakes showed that targeted and tailored interventions to parents such as reminders and education were effective in increasing paediatric vaccine uptake. A local randomised controlled trial showed a multiple-component intervention package (including key influenza information with vaccination reminders) targeting postpartum mothers could improve influenza vaccine uptake in Hong Kong children.

**Added value of this study**

This randomised controlled trial showed that a multiple-component intervention package increased rotavirus vaccine uptake by 1.7 times or by 33 percent-points. In particular, removal of the financial barrier increased uptake by 1.46 times or 25 percent-points. Also, this study confirmed the effectiveness of educational information and vaccination reminders to increase paediatric vaccine uptake, and specifically revealed these worked in rotavirus vaccination decision (uptake increased by 1.17 times or 8 percent-points).

**Implications of all the available evidence**

A multiple-component intervention package, and in particular providing free vaccine, could increase rotavirus vaccine uptake in Hong Kong children. The impact of intervention package was greatest in low-income families, emphasising the importance of removing financial barriers to vaccination to promote equity. This may also apply in other similar settings in the region or globally.

## Introduction

Rotavirus is a major cause of morbidity and mortality globally, both in developed and developing countries. Almost every child will be infected with rotavirus by the age of 5 years irrespective of where they live, and 35%–40% of diarrheal hospitalisations in this age group are due to rotavirus.^1^ Among this age group, there were 1.8 million and 333,000 rotavirus hospitalisations globally and in Western Pacific respectively in 2019,^2^ and the cumulative risk of rotavirus hospitalisation in Hong Kong was 1 in 33 children based on a disease burden study from 1997 to 2011.^3^

With the severe burden of rotavirus, there is an increasing number of countries (124 countries globally and 15 countries in the Western Pacific) that have introduced rotavirus vaccine into their national immunisation programmes as of May 2024.^4^ Children in Hong Kong are provided with free vaccinations against 12 antigens under the Hong Kong Government's Childhood Immunisation Programme (CIP). Vaccines not included in the routine CIP, such as rotavirus vaccine, can be obtained if parents pay out-of-pocket and take their children to doctors in the private sector. Rotavirus vaccine has been shown to be 92%–96% effective against hospitalisation^5^ and is likely to be cost-saving and have a favourable benefit–risk profile in Hong Kong.^6^ Although rotavirus vaccine has been licensed in Hong Kong since 2006, its uptake remains low (33.3% in 2015 based on unpublished data from a published study^7^). This compares to the high immunisation coverage rates of vaccines included in the CIP of over 95%.^8^ A knowledge, attitudes and practices study of 500 mother–infant pairs conducted in Hong Kong in 2014 showed that household income was associated with both perceived benefits of rotavirus vaccine and self-efficacy to vaccinate with the highest income quartile being 1.5 times more likely to vaccinate their children with rotavirus vaccine.^9^

In view of the relatively low uptake of this safe, effective and cost-saving vaccine, and the very significant local disease burden, this randomised controlled trial (RCT) examined whether (i) removal of financial barriers and/or (ii) provision of key information on rotavirus with vaccination reminders can increase rotavirus vaccine uptake in Hong Kong children.

## Methods

### Study design

This RCT was conducted in Hong Kong with participants recruited at two public hospitals (Prince of Wales Hospital situated in the New Territories Eastern Cluster and the United Christian Hospital situated in the Kowloon East Cluster, with a catchment of approximately 20% of all Hong Kong births). Ethics approval was granted by the Joint Chinese University of Hong Kong—New Territories East Cluster Clinical Research Ethics Committee (Ref.: CRE-2019.718-T) and the Research Ethics Committee (Kowloon Central/Kowloon East) (Ref.: KC/KE-20-0010/ER-2).

### Participants

Postpartum mothers were recruited from the postnatal wards of two public hospitals from 16 February to 30 July 2021. All postpartum mothers on the wards present on the postnatal wards during the recruitment period were approached. Mothers were asked for their interest in participating in the study and checked for their eligibility to be included in the study according to the inclusion criteria. A convenience sample of women were invited to participate in this prospective RCT if they met the following inclusion criteria: (i) postpartum mother aged 18 years or above; (ii) Cantonese speaking and able to read traditional Chinese; (iii) had a plan to remain in Hong Kong with the infant after birth during the study period; (iv) no obvious cognitive abnormality; (v) no serious obstetric complications; and (vi) baby is full-term (≥37 weeks of gestation) with no congenital abnormalities. If an enrolled mother had a multiple birth, only data for the elder child was included in the analyses. Written consent was obtained if the mothers agreed to participate.

### Sample size calculation

The provision of publicly available standard information about rotavirus infection to all participants may increase their knowledge of the vaccine in subjects who do not know the vaccine before joining the study. To ensure the sample size is enough to examine the study objectives, we estimated the rotavirus vaccine uptake will be increased from 40% to at least 55%. Anticipating 20% drop-out, based on a required power of 80% and a significance level of 0.05, by using G∗Power version 3.1.9.2, the sample size required in each group was 217, and 651 in total.

### Randomisation and masking

After consenting, all subjects were randomly allocated into three groups (either the control or two intervention groups) using block randomisation,^10^ with each group having similar numbers of participants. The intervention allocation in random block sizes of six to twelve was randomly generated using statistical software R (version 4.2.3), with the block sizes kept unknown to investigators and research staff delivering treatments and carrying out interviews. The allocation sequence was saved in individual electronic files. The research staff member coordinating the interventions opened the individual electronic files one by one before intervention delivery and was not involved in the final data collection. Group allocation was concealed to the investigators, participants, and the research staff coordinating the final data collection.

### Intervention and control

#### Control group

Subjects in the control group received the publicly available, standard information from the Centre for Health Protection about rotavirus infection by post three to five days after enrolment (Appendix 1).

#### Intervention group 1

**Fig. 1:**
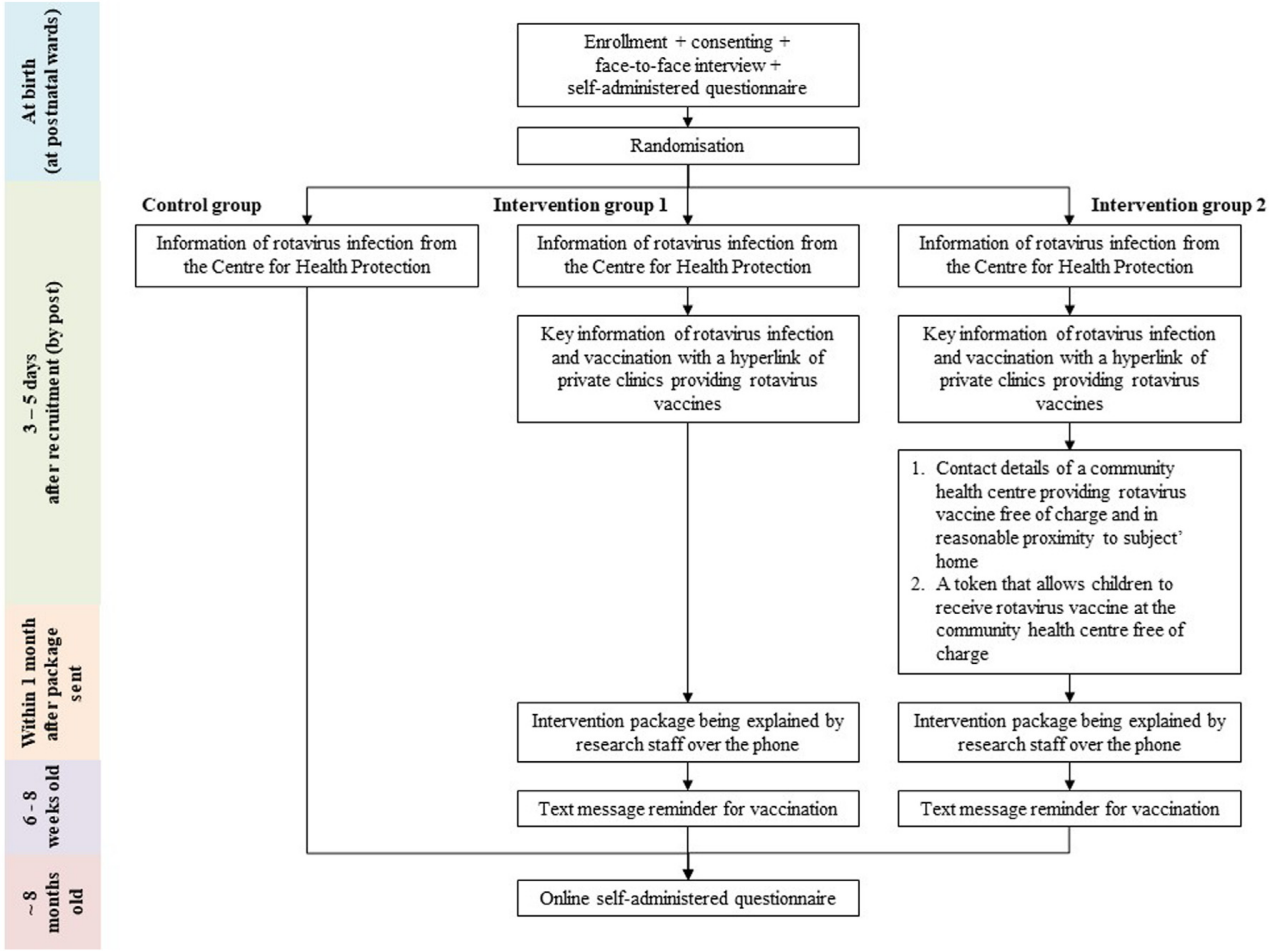
**Study logistic flow**.

Subjects in the intervention group 1 received the same standard information about rotavirus infection as the control-subjects by post three to five days after enrolment, and an additional researcher-designed intervention information sheet (Appendix 2) containing (i) key information about the risks of rotavirus to children and the benefits of rotavirus vaccination; (ii) eligible age to receive rotavirus vaccine; (iii) a hyperlink to a government webpage showing clinics in the private sector that provide rotavirus vaccines; and (iv) guidance on searching these clinics. A text message reminder for vaccination with the same hyperlink to search for private clinics was sent at the child's age of six to eight weeks. Fig. 1 shows the logistic flow of this RCT.

#### Intervention group 2

Subjects in the intervention group 2 received the same standard information about rotavirus infection as the control-subjects by post three to five days after enrolment, and the same key information as the intervention group 1. Additionally, they received contact details of a specific community health centre within the United Christian Nethersole Community Health Service (UCN) in reasonable proximity to their home (Appendix 3); and tokens to receive rotavirus vaccines free of charge at the specific UCN clinics (Appendix 4). A text message reminder for vaccination with contact details of the specific UCN clinic and the hyperlink to search for private clinics was sent at the child's age of six to eight weeks.

For both intervention groups, we attempted to explain the intervention packages over the phone within a month after the package had been sent. If the intervention-subjects did not answer the phone after two trials, text messages were sent to confirm they received the intervention packages and make appointments to explain the package over the phone. If explanation over the phone of how to use the tokens for free rotavirus vaccination failed, then this was explained through text messages to mothers in intervention group 2.

### Data collection and outcomes

Data were collected at two time-points: face-to-face interviews followed by self-administered questionnaires at enrolment on the postnatal wards; and online self-administered questionnaires when the children were approximately eight months old. Key demographic information and plans to give rotavirus vaccine were collected in the baseline questionnaire. At approximately eight months old, the child's rotavirus vaccination status was collected and subjects were asked to provide an electronic copy of the children's vaccination records. At both time-points, subjects were asked about their attitudes towards rotavirus vaccine with the same set of questions.

Maternal attitudes in relation to rotavirus and its vaccine were assessed by a questionnaire based on the Health Belief Model.^11^^,^^12^ Some questionnaire items were adapted from a previous RCT investigating increasing influenza vaccine uptake,^13^ and some were newly designed. The draft questionnaire was pilot-tested in 20 mothers at the postnatal wards of one study hospital to ensure good understanding and consistency of questionnaire items. The questionnaires were then revised based on the results from the pilot.

### Statistical analyses

Consolidated Standards of Reporting Trials (CONSORT) 2010 statement^14^ was followed in the reporting of this study. Intention-to-treat analysis^15^ was used and any missing rotavirus vaccination status was taken as no vaccination. Rotavirus vaccination uptake was calculated from both mothers’ self-report and the immunisation records provided by mothers and UCN.

Descriptive statistics of the demographic variables in the three groups were calculated. To assess the usefulness of the interventions in increasing rotavirus vaccine uptake, we performed chi-squared tests to compare rotavirus vaccine uptakes between control and intervention groups, and calculated the relative risks (RR) with 95% confidence intervals (CI) for the relative likelihood to give the vaccine in different groups. To determine the effectiveness of the full intervention package to increase uptake of rotavirus vaccine in children, intervention group 2 was compared to the control group. To determine whether removal of financial barriers can increase uptake of rotavirus vaccine in children, intervention group 2 was compared with the intervention group 1. To determine whether provision of key information on rotavirus infection and vaccination with vaccination reminders can increase uptake of rotavirus vaccine in children, intervention group 1 was compared with the control group. The effectiveness of the interventions was also assessed among subjects with different monthly household income and plan to give rotavirus vaccine during the immediate postpartum period. Each attitude statement was scored from 1 to 5 (1 = strongly disagree; 2 = disagree; 3 = don't know; 4 = agree; and 5 = strongly agree) with a higher score meaning greater agreement with the statement. Permutation tests^16^ were conducted on the paired data to determine any difference in attitude scores before and after receiving the interventions. All statistical analyses were performed using statistical software R (version 4.2.3) and a two-tailed P-value <0.05 was taken as statistically significant. This RCT has been registered in the Chinese Clinical Trial Register (Ref.: ChiCTR2000039791).

### Role of the funding source

This work was supported by the Health and Medical Research Fund by the Health Bureau, Government of Hong Kong SAR [Ref.: 19180202]. The funder of the study had no role in study design, data collection, data analysis, data interpretation, or writing of the report.

## Results

### Recruitment and subjects overview

**Fig. 2:**
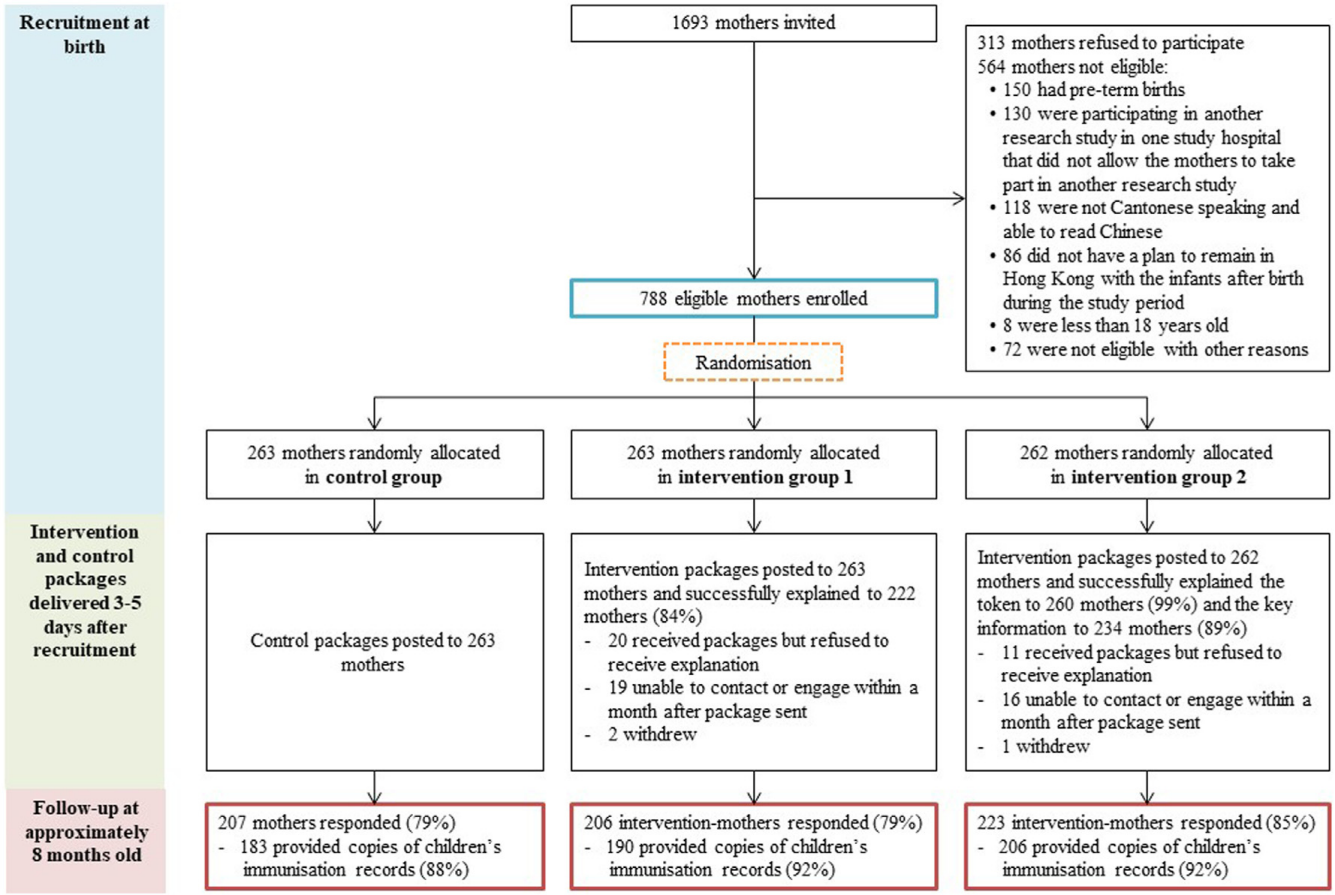
**Subject flow chart of the study**.

From 16 February to 30 July 2021, 1693 postpartum mothers were invited to participate, of which 788 eligible mothers (70% of the 1129 eligible mothers) were enrolled and randomly allocated into control group (n = 263), intervention group 1 (n = 263) and intervention group 2 (n = 262) (Fig. 2). Control and intervention packages were sent to the respective groups three to five days after enrolment. The information sheet in the intervention packages was explained to 222 (84%) and 234 (89%) mothers in the intervention groups 1 and 2 respectively. Almost all (99%) mothers in the intervention group 2 received an explanation about the use of tokens for free rotavirus vaccination. When the children reached the age of approximately eight months, 636 mothers (81%) responded to the online questionnaires.

Table 1 presents the descriptive statistics of subjects’ background information, awareness of rotavirus vaccine, and plan to vaccinate their children with rotavirus vaccine collected at enrolment, and their infant feeding methods and disease history obtained at the end of the study.

**Table 1:** Baseline information, infant feeding methods and disease history of mother–infant pairs in intervention group 2, intervention group 1, and control group.

	Intervention group 2 n (%)	Intervention group 1 n (%)	Control group n (%)
**Collected postpartumly**			
Background			
Sex of newborns			
Male	139 (53)	114 (43)	122 (46)
Female	123 (47)	149 (57)	141 (54)
Birth order			
1	111 (42)	125 (48)	129 (49)
More than 1	151 (58)	138 (52)	134 (51)
Birthing hospital			
Prince of Wales Hospital	154 (59)	150 (57)	155 (59)
United Christian Hospital	108 (41)	113 (43)	108 (41)
Mothers' age			
18–27	35 (13)	28 (11)	28 (11)
28–32	76 (29)	91 (35)	79 (30)
33–37	103 (39)	119 (45)	114 (43)
38–47	48 (18)	25 (10)	42 (16)
Mothers' highest educational level			
Junior secondary or below	25 (10)	23 (9)	23 (9)
Senior secondary	84 (32)	86 (33)	83 (32)
Matriculation or non-degree course	28 (11)	29 (11)	30 (11)
Associate degree/higher diploma	31 (12)	34 (13)	40 (15)
University degree or above	94 (36)	91 (35)	87 (33)
Mothers' place of birth			
Hong Kong	161 (61)	158 (60)	162 (62)
China	96 (37)	101 (38)	101 (38)
Other	5 (2)	4 (2)	0 (0)
Monthly household income (HKD)			
<30,000	63 (24)	76 (29)	61 (23)
30,000–39,999	63 (24)	50 (19)	58 (22)
40,000–49,999	46 (18)	46 (17)	47 (18)
≥50,000	81 (31)	82 (31)	79 (30)
Vaccination			
Heard of rotavirus vaccine before	185 (71)	166 (63)	185 (70)
Rotavirus vaccination plan during immediate postpartum period			
Yes	142 (54)	131 (50)	145 (55)
No	14 (5)	21 (8)	17 (6)
Unsure	106 (40)	111 (42)	101 (38)
**Collected at approximately children's age of 8 months**			
Ever breastfed	201 (90)	178 (87)	182 (88)
Disease history			
Number of times visited a health centre, a general practice clinic or hospital outpatient department for a reason other than vaccination			
0 times	86 (39)	79 (39)	73 (35)
1–2 times	90 (40)	88 (43)	95 (46)
More than 3 times	47 (21)	38 (19)	38 (18)
Number of times stayed overnight in a hospital for medical care			
0 times	143 (64)	119 (58)	129 (62)
1–2 times	70 (31)	78 (38)	68 (33)
More than 3 times	10 (4)	9 (4)	10 (5)
Ever contracted any gastrointestinal infection	19 (9)	19 (9)	16 (8)
Admitted to hospital overnight for the gastrointestinal infection	3 (16)	4 (21)	2 (13)

### Effectiveness of intervention packages

To be conservative, missing rotavirus vaccination status was taken as unvaccinated. 81% (212/262), 56% (146/263) and 48% (125/263) children from the intervention group 2, intervention group 1 and control group respectively received rotavirus vaccines (Table 2). Although not statistically significant, intervention 1 (key information with vaccination reminders) increased uptake by 8 percent-points (from 48% to 56%, P-value = 0.07). Subjects receiving key rotavirus information with vaccination reminders were 1.17 times (95% CI of RR = 0.99–1.38) more likely to give rotavirus vaccine to their children, compared to those receiving only public information. Removal of financial barriers increased uptake by an additional 25 percent-points (from 56% to 81%, P-value <0.0001), i.e., the whole intervention package increased uptake by 33 percent-points (from 48% to 81%) compared with the control group receiving only publicly available standard information. Subjects provided with tokens for free rotavirus vaccination were 1.46 times (95% CI of RR = 1.29–1.66) more likely to give rotavirus vaccine to their children, compared to those only receiving key information with reminders; and were 1.7 times (95% CI of RR = 1.49–1.97) more likely to give the vaccine to their children, compared to those receiving only publicly available standard information.

**Table 2:** Rotavirus vaccine uptakes between intervention group 2, intervention group 1, and control group.

	Intervention group 2 n (%)	Intervention group 1 n (%)	Control group n (%)	Absolute difference (Intervention 2–control) (%)	P-value	Absolute difference (Intervention 2–intervention 1) (%)	P-value	Absolute difference (Intervention 1–control) (%)	P-value
Unvaccinated for missing vaccination status	212/262 (81)	146/263 (56)	125/263 (48)	33	<0.001∗	25	<0.001∗	8	0.07
Vaccination status available	212/223 (95)	146/206 (71)	125/207 (60)	35	<0.001∗	24	<0.001∗	10	0.02∗

∗P-value <0.05.

At enrolment, 53% of mothers had a plan to vaccinate their children with rotavirus vaccine, 40% were unsure and 7% did not have a plan to vaccinate. Effects of the intervention packages were also examined among mothers with different rotavirus vaccination plans at the immediate postpartum period (Table 3). Removal of financial barriers significantly increased rotavirus vaccine uptakes among mothers who had a plan to and were unsure whether to give the vaccine at the immediate postpartum period (P-value <0.0001). The full intervention significantly increased rotavirus vaccine uptake in all groups.

**Table 3:** Rotavirus vaccine uptakes by postpartum vaccination plan.

At enrolment, mothers had a plan to give rotavirus vaccine to their children	Vaccinated n (%)			Absolute difference (Intervention 2–control) (%)	P-value	Absolute difference (Intervention 2–intervention 1) (%)	P-value	Absolute difference (Intervention 1–control) (%)	P-value
	Intervention group 2	Intervention group 1	Control group						
Yes	129 (96)	92 (84)	92 (80)	16	<0.001∗	13	<0.001∗	4	0.29
No	11 (92)	9 (53)	2 (15)	76	<0.001∗	39	0.08	38	0.08
Unsure	72 (81)	45 (57)	31 (39)	42	<0.001∗	24	<0.001∗	18	0.18

∗P-value <0.05.

**Fig. 3:**
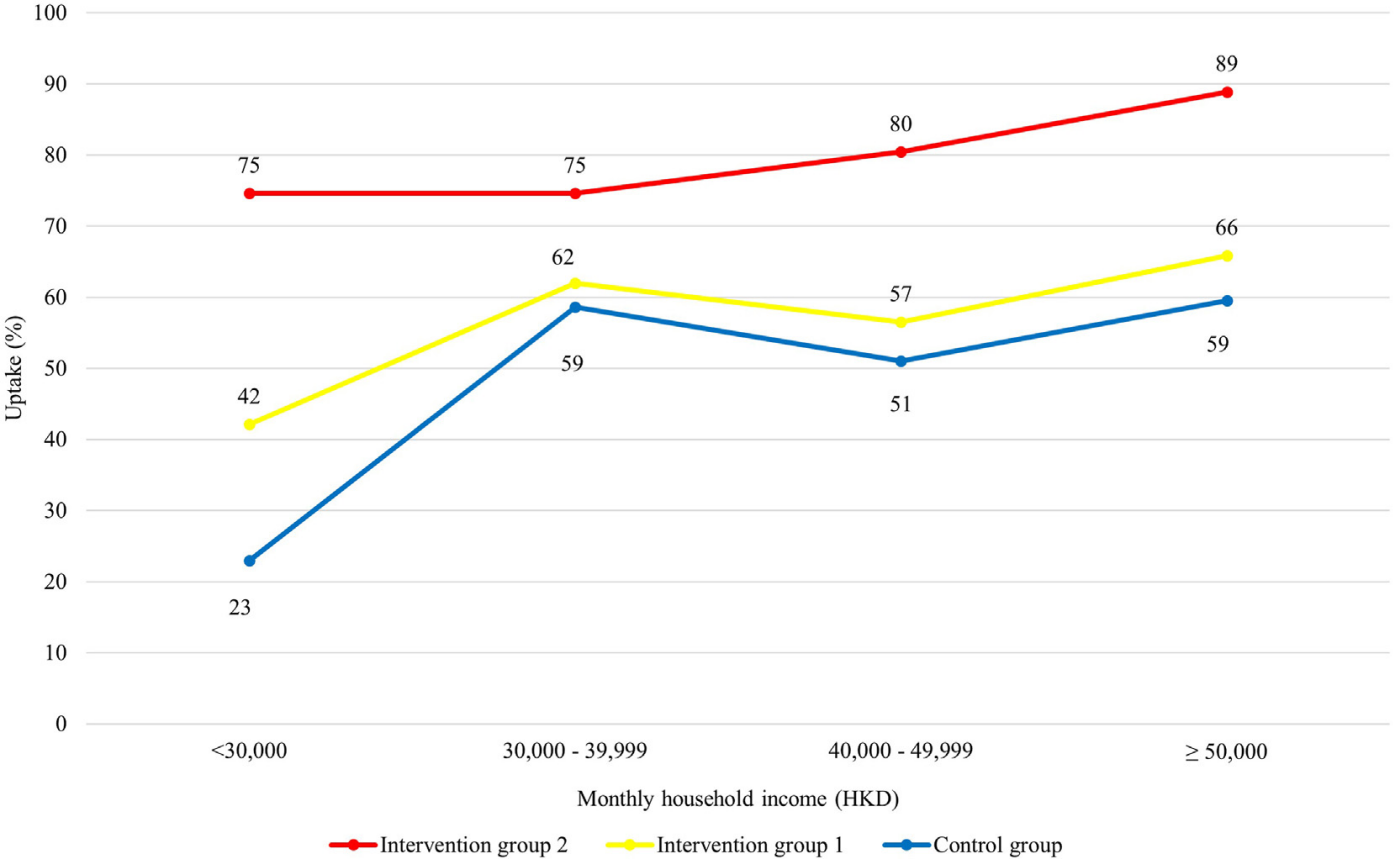
**Rotavirus vaccine uptakes by monthly household income between groups**.

**Fig. 4:**
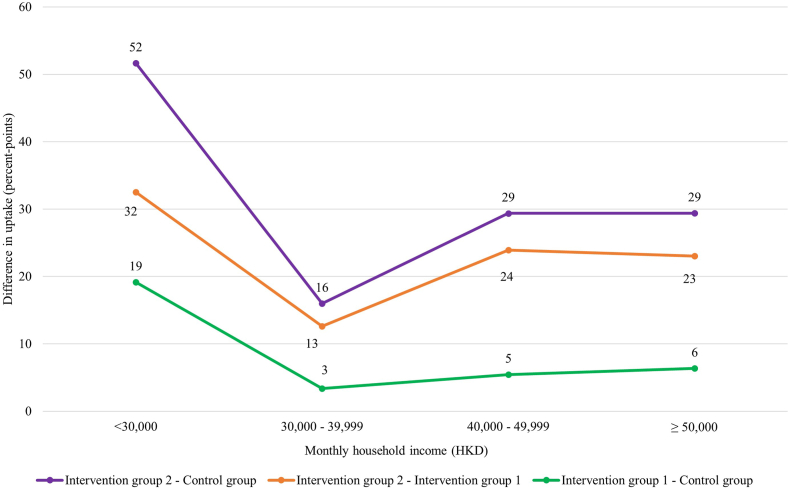
**Difference in rotavirus vaccine uptakes by monthly household income between groups**.

Additionally, we investigated the effectiveness of the intervention packages among mothers with different monthly household income (Fig. 3). Provision of key rotavirus information with a vaccination reminder only significantly improved vaccination uptakes in children in families with less than HKD 30,000 monthly household income, but not the other income groups (Fig. 4). Removal of financial barriers significantly improved uptakes in groups of less than HKD 30,000, HKD 40,000–49,999 and more than or equal to HKD 50,000, but not the group of HKD 30,000–39,999 (Fig. 4).

### Intervention components

At the end of the study, participants were asked if their decision to give rotavirus vaccine to their children was influenced by the study. 50% of intervention group 2, 44% of intervention group 1 and 33% of control group mothers perceived their decisions were influenced. Subjects in the intervention group 2 thought the tokens for free vaccination was the most important intervention component influencing their vaccination decision. Subjects in both intervention groups perceived the key rotavirus information was important on their decisions, followed by the text message reminders for vaccination. The guidance on searching clinics providing rotavirus vaccine and the explanation on information by phone were perceived as less important.

### Perceived factors of rotavirus vaccination decision

**Fig. 5:**
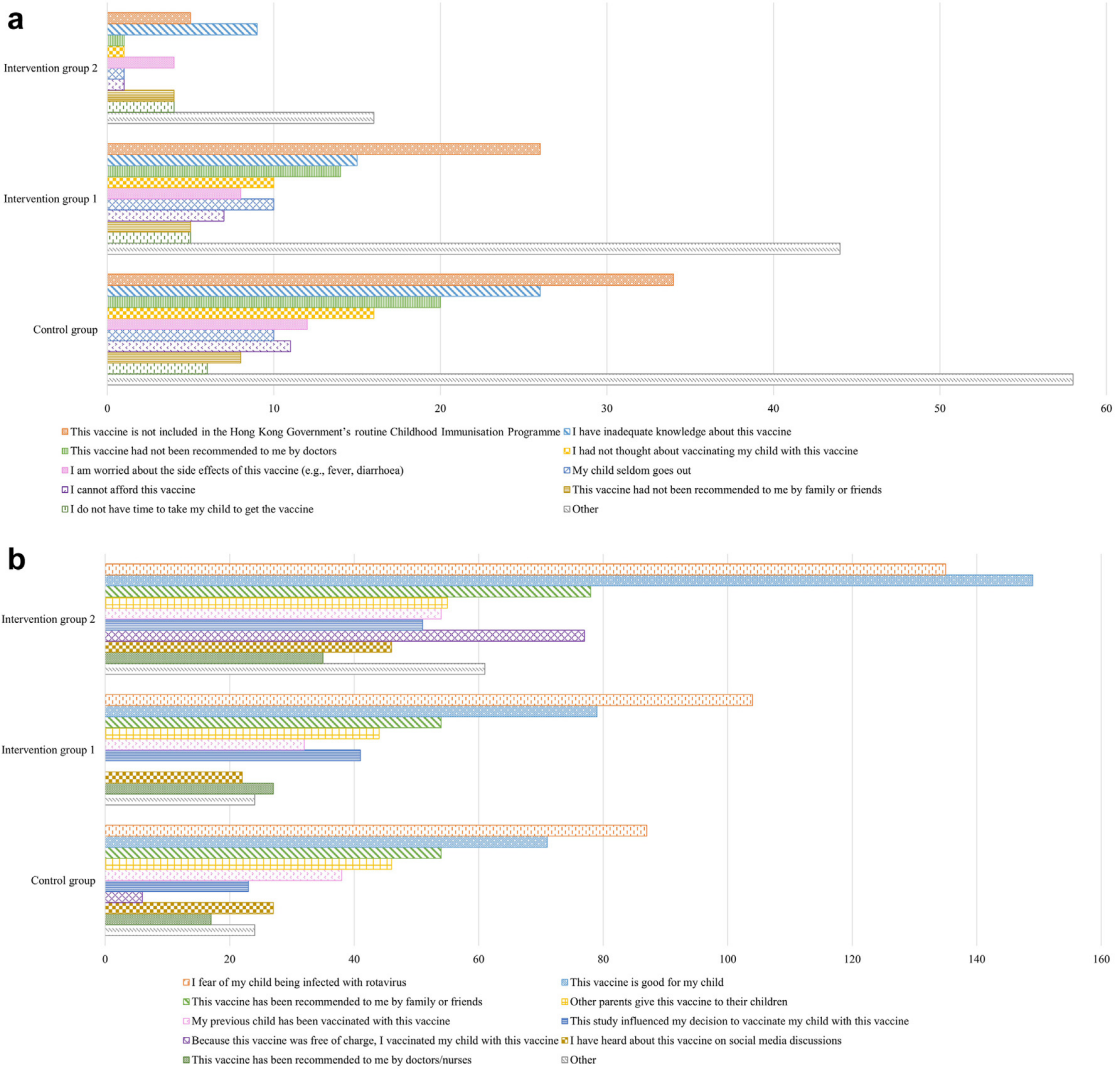
**Mothers' perceived factors of rotavirus vaccination decision**. **a. Reasons for not giving rotavirus vaccine. b. Reasons for giving rotavirus vaccine**.

Without removal of the financial barrier for rotavirus vaccination, subjects in intervention group 1 and control group did not vaccinate their children with rotavirus vaccine mainly because the vaccine is not included in the routine CIP, followed by inadequate knowledge about the vaccine and it not being recommended by doctors (Fig. 5a). For intervention group 2, the main reason for not giving rotavirus vaccine was inadequate knowledge of the vaccine. For the mothers who vaccinated their children with rotavirus vaccine, the reasons for vaccination in intervention group 1 and the control group were similar (Fig. 5b). The most important reason for vaccination in intervention group 2 was “the vaccine is good for my child”, which was ranked second in the other two groups.

### Maternal attitudes towards rotavirus infection and vaccination before and after intervention

Mothers' agreement to statements about attitudes towards rotavirus infection and rotavirus vaccination at the beginning and the end of the study were compared (Table 4). In general, subjects in all groups perceived rotavirus as more severe and rotavirus vaccine as more beneficial when their children were approximately eight months old compared to the immediate postpartum period. Husband's recommendation, news reports and other mothers' decision to vaccinate their children became more important factors when they decided whether to vaccinate their children with rotavirus vaccine.

**Table 4:** Maternal attitudes towards rotavirus infection and vaccination before and after treatments.

	Intervention group 2				Intervention group 1				Control group			
	Score before treatments mean ± SD	Score after treatments mean ± SD	Score difference mean ± SD	P-value	Score before treatments mean ± SD	Score after treatments mean ± SD	Score difference mean ± SD	P-value	Score before treatments mean ± SD	Score after treatments mean ± SD	Score difference mean ± SD	P-value
**Perceived susceptibility**												
My child is healthy so I am not concerned about him/her getting rotavirus	2.45 ± 1.01	2.39 ± 1.14	−0.01 ± 1.27		2.44 ± 0.97	2.59 ± 1.15	0.23 ± 1.23	∗	2.38 ± 0.96	2.59 ± 1.15	0.19 ± 1.17	∗
Children aged below 5 years are likely to get rotavirus infection	3.64 ± 0.76	4.22 ± 0.72	0.54 ± 0.84	∗	3.67 ± 0.78	4.08 ± 0.74	0.36 ± 0.92	∗	3.78 ± 0.74	4.02 ± 0.75	0.19 ± 1.17	∗
Rotavirus is an uncommon virus so my child will not easily get it	2.36 ± 0.8	2.13 ± 0.92	−0.19 ± 1.1	∗	2.39 ± 0.78	2.32 ± 1.01	0.04 ± 0.96		2.28 ± 0.81	2.43 ± 0.99	0.15 ± 0.9	∗
Everyone may get rotavirus	3.78 ± 0.77	4.08 ± 0.79	−0.19 ± 1.1	∗	3.81 ± 0.74	4.04 ± 0.78	0.04 ± 0.96	∗	3.73 ± 0.87	3.9 ± 0.82	0.15 ± 1.03	
In general, a healthy adult 70 years old is more likely to get rotavirus than a healthy child below 5 years old	2.8 ± 0.68	2.86 ± 0.92	−0.19 ± 1.1		2.8 ± 0.72	2.8 ± 0.83	0.04 ± 0.96		2.79 ± 0.71	2.81 ± 0.8	0.15 ± 1.03	
**Perceived severity**												
In general, rotavirus diarrhoea is usually more serious in a healthy adult 70 years old than in a healthy child below 5 years old	2.87 ± 0.67	2.9 ± 0.87	0.06 ± 0.9		2.89 ± 0.7	2.94 ± 0.85	0.1 ± 0.89		2.89 ± 0.69	2.87 ± 0.79	−0.01 ± 1	
Rotavirus diarrhoea has serious health consequences on a child	3.96 ± 0.81	4.42 ± 0.78	0.43 ± 0.94	∗	3.98 ± 0.81	4.33 ± 0.8	0.31 ± 1.03	∗	4.01 ± 0.77	4.21 ± 0.82	0.14 ± 0.94	∗
Rotavirus diarrhoea is a light illness	2.58 ± 0.88	2.7 ± 1.26	0.15 ± 1.3		2.56 ± 0.88	2.78 ± 1.19	0.31 ± 1.36	∗	2.56 ± 0.91	2.64 ± 1.16	0.12 ± 1.18	
Rotavirus infection can sometimes be serious enough that a child needs to be admitted to the hospital	3.94 ± 0.87	4.49 ± 0.66	0.51 ± 0.93	∗	3.91 ± 0.85	4.39 ± 0.77	0.39 ± 0.98	∗	3.98 ± 0.8	4.36 ± 0.74	0.33 ± 0.89	∗
Rotavirus infection can sometimes be serious enough to cause death in a child	3.71 ± 0.83	4.34 ± 0.79	0.58 ± 1	∗	3.67 ± 0.88	4.19 ± 0.89	0.45 ± 1.06	∗	3.78 ± 0.84	4.09 ± 0.91	0.28 ± 0.98	∗
If my child gets rotavirus, the disease can spread and cause illness to other family members	3.49 ± 0.71	3.72 ± 1	0.23 ± 1.05	∗	3.48 ± 0.85	3.64 ± 0.96	0.11 ± 1.05		3.52 ± 0.74	3.73 ± 0.91	0.18 ± 0.94	∗
**Perceived benefit**												
Rotavirus vaccines are safe for children	3.73 ± 0.73	4.26 ± 0.61	0.47 ± 0.77	∗	3.71 ± 0.71	4.16 ± 0.61	0.38 ± 0.71	∗	3.76 ± 0.7	4.06 ± 0.71	0.27 ± 0.8	∗
Rotavirus vaccine can prevent convulsions or seizures in children	3.66 ± 0.69	4.2 ± 0.7	0.5 ± 0.88	∗	3.63 ± 0.71	4.07 ± 0.73	0.4 ± 0.88	∗	3.63 ± 0.73	4.04 ± 0.75	0.37 ± 0.76	∗
Rotavirus vaccine can reduce risk of hospitalisation in children	3.77 ± 0.69	4.21 ± 0.71	0.42 ± 0.88	∗	3.7 ± 0.75	4.13 ± 0.69	0.4 ± 0.84	∗	3.73 ± 0.74	4.1 ± 0.67	0.3 ± 0.75	∗
Rotavirus vaccine can reduce risk of death in children	3.69 ± 0.74	4.23 ± 0.72	0.51 ± 0.88	∗	3.69 ± 0.77	4.1 ± 0.72	0.37 ± 0.83	∗	3.7 ± 0.75	4.04 ± 0.76	0.29 ± 0.78	∗
Rotavirus vaccinations effectively protect against rotavirus diarrhoea in children	3.83 ± 0.67	4.31 ± 0.64	0.43 ± 0.73	∗	3.82 ± 0.73	4.14 ± 0.72	0.27 ± 0.84	∗	3.86 ± 0.74	4.14 ± 0.65	0.25 ± 0.73	∗
**Perceived barrier**												
Rotavirus vaccine can cause rotavirus in some people	2.9 ± 0.76	3.15 ± 1.15	0.26 ± 1.32	∗	2.86 ± 0.78	3.17 ± 1.06	0.35 ± 1.16	∗	2.86 ± 0.81	3.15 ± 1.05	0.28 ± 1.3	∗
It is better for a child to develop immunity by getting rotavirus rather than getting a vaccine to prevent rotavirus infection	2.84 ± 0.92	2.87 ± 1.21	0.06 ± 1.31		2.78 ± 0.92	3.04 ± 1.18	0.34 ± 1.28	∗	2.69 ± 0.93	3.07 ± 1.13	0.39 ± 1.27	∗
Too many vaccines can overwhelm a child's immune system	2.57 ± 0.86	2.55 ± 1.05	0.04 ± 1.13		2.67 ± 0.86	2.79 ± 1.02	0.24 ± 1.08	∗	2.53 ± 0.84	2.75 ± 1.03	0.23 ± 1.07	∗
It is difficult for me to find the time to take my child for rotavirus vaccinations	1.99 ± 0.74	2.12 ± 1.06	0.19 ± 1.13	∗	2.1 ± 0.78	2.26 ± 1.13	0.22 ± 1.12	∗	1.99 ± 0.77	2.29 ± 1.1	0.32 ± 1.12	∗
If rotavirus vaccine is free of charge, I will vaccinate my child with rotavirus vaccine	3.92 ± 1.03	4.25 ± 0.9	0.27 ± 1.07	∗	3.86 ± 1.01	4.29 ± 0.91	0.38 ± 1.2	∗	4 ± 0.99	4.22 ± 0.91	0.14 ± 1.09	
When deciding whether or not to vaccinate my child with rotavirus vaccine, the following factors are possible reasons why I may not give the vaccine:												
If the vaccine costs a lot of money	3.04 ± 1.21	3.42 ± 1.2	0.37 ± 1.29	∗	3.12 ± 1.18	3.53 ± 1.18	0.36 ± 1.38	∗	2.98 ± 1.24	3.43 ± 1.2	0.46 ± 1.25	∗
If there are possible side effects of vaccination	4.13 ± 0.83	4.13 ± 0.87	−0.06 ± 1		4.12 ± 0.79	4.04 ± 0.93	−0.13 ± 1		4.08 ± 0.89	4.14 ± 0.81	0.01 ± 0.95	
If there is negative news about the vaccine	4.18 ± 0.83	4.06 ± 0.91	−0.18 ± 1.03	∗	4.1 ± 0.85	4.05 ± 0.93	−0.13 ± 1.11		4.06 ± 0.92	4.12 ± 0.85	0.02 ± 0.96	
If the vaccine is not given as part of the Hong Kong Government's routine Childhood Immunisation Programme	3.11 ± 1.16	3.11 ± 1.15	0.06 ± 1.41		3.19 ± 1.12	3.1 ± 1.21	−0.07 ± 1.32		2.96 ± 1.06	3.03 ± 1.12	0.08 ± 1.28	
If there is a lack of conveniently located clinics that provide rotavirus vaccinations	2.85 ± 1.14	2.94 ± 1.23	0.16 ± 1.42		2.95 ± 1.08	3 ± 1.21	0.17 ± 1.35		2.77 ± 1.07	3 ± 1.17	0.21 ± 1.36	∗
If an appointment at the clinic to receive the vaccine is required	2.42 ± 1.01	2.59 ± 1.12	0.23 ± 1.21	∗	2.4 ± 0.91	2.65 ± 1.14	0.33 ± 1.16	∗	2.41 ± 0.94	2.59 ± 1.12	0.16 ± 1.19	
If the vaccine requires too many doses	3.02 ± 1.14	3.07 ± 1.15	0.05 ± 1.44		3 ± 1.06	3.16 ± 1.17	0.24 ± 1.3	∗	2.74 ± 1.07	3.08 ± 1.18	0.27 ± 1.31	∗
If the vaccine is administrated by mouth	2.56 ± 1.03	2.55 ± 1.12	0.09 ± 1.24		2.62 ± 0.95	2.73 ± 1.15	0.2 ± 1.2	∗	2.5 ± 1	2.73 ± 1.16	0.22 ± 1.3	∗
**Self-efficacy**												
I am confident to make the best decision about vaccinating my child with rotavirus vaccine	3.87 ± 0.77	4.34 ± 0.72	0.45 ± 0.96	∗	3.91 ± 0.7	4.32 ± 0.71	0.39 ± 0.85	∗	3.92 ± 0.75	4.08 ± 0.81	0.11 ± 0.9	
I have access to the information I need to make good decisions about vaccinating my child with rotavirus vaccine	3.28 ± 1.01	4.14 ± 0.77	0.87 ± 1.19	∗	3.32 ± 1	4.07 ± 0.84	0.79 ± 1.19	∗	3.3 ± 1	3.71 ± 0.98	0.43 ± 1.21	∗
I am confident that I am able to afford rotavirus vaccine	3.75 ± 0.8	3.97 ± 0.98	0.16 ± 1.03	∗	3.82 ± 0.83	4 ± 1	0.13 ± 1.11		3.8 ± 0.87	3.9 ± 1.01	0.06 ± 1.19	
I am confident to take my child to receive all doses of rotavirus vaccine on schedule	3.9 ± 0.75	4.18 ± 0.86	0.22 ± 0.94	∗	3.9 ± 0.81	4.18 ± 0.87	0.19 ± 1.02	∗	3.98 ± 0.77	4.03 ± 0.97	−0.01 ± 1.08	
**Cue to action**												
Doctors' recommendation is an important factor when deciding whether or not to vaccinate my child with rotavirus vaccine	4.03 ± 0.75	4.18 ± 0.78	0.11 ± 0.94		3.96 ± 0.83	4 ± 0.91	0 ± 1.02		4.02 ± 0.8	3.91 ± 1.03	−0.11 ± 1.15	
Nurses' recommendation is an important factor when deciding whether or not to vaccinate my child with rotavirus vaccine	3.81 ± 0.88	4 ± 0.89	0.13 ± 1.03		3.76 ± 0.88	3.85 ± 0.98	0.05 ± 1.09		3.79 ± 0.94	3.7 ± 1.07	−0.13 ± 1.19	
My husband's recommendation is an important factor when deciding whether or not to vaccinate my child with rotavirus vaccine	3.7 ± 0.97	3.88 ± 0.98	0.18 ± 1.14	∗	3.54 ± 1	3.85 ± 0.95	0.31 ± 1.16	∗	3.61 ± 0.97	3.79 ± 1.01	0.17 ± 1.2	∗
My family's recommendation is an important factor when deciding whether or not to vaccinate my child with rotavirus vaccine	3.64 ± 0.96	3.72 ± 1.02	0.07 ± 1.17		3.53 ± 0.98	3.75 ± 1.01	0.2 ± 1.17	∗	3.56 ± 0.97	3.69 ± 1.04	0.12 ± 1.24	
My friends' recommendation is an important factor when deciding whether or not to vaccinate my child with rotavirus vaccine	3.36 ± 1.03	3.66 ± 1	0.27 ± 1.18	∗	3.34 ± 1.02	3.51 ± 1.08	0.16 ± 1.17		3.4 ± 0.99	3.55 ± 1.03	0.16 ± 1.26	
If most of the mothers I know vaccinate their children against rotavirus, I will vaccinate my child too	3.61 ± 1	3.97 ± 0.96	0.33 ± 1.23	∗	3.62 ± 0.93	3.83 ± 1.01	0.19 ± 1.14	∗	3.57 ± 0.97	3.8 ± 1.03	0.18 ± 1.23	∗
If most people important to me think I should get my child vaccinated for rotavirus, I will do so	3.71 ± 0.96	4.01 ± 0.95	0.27 ± 1.17	∗	3.67 ± 0.93	3.91 ± 0.96	0.23 ± 1.1	∗	3.7 ± 0.9	3.82 ± 1.02	0.1 ± 1.19	
If there is a news report about a child being seriously ill or dying of rotavirus infection in Hong Kong, I will vaccinate my child with rotavirus vaccine	3.5 ± 1.05	4 ± 1.01	0.46 ± 1.31	∗	3.49 ± 1.07	3.92 ± 1.07	0.45 ± 1.29	∗	3.45 ± 1.06	3.86 ± 1.14	0.38 ± 1.43	∗

∗P-value <0.05.

At the end of the study, subjects in intervention group 1 and the control group were more concerned about a vaccine that requires too many doses and that is administrated by mouth, and too many vaccines could overwhelm a child's immune system, and preferred more on developing immunity by getting rotavirus than getting a vaccine to prevent rotavirus infection. Subjects in both intervention groups agreed, at the end of the study, that they were more likely to give rotavirus vaccine to their children if the vaccine is free of charge, less likely to give if an appointment at the clinic is needed, and more confident to make the best decision about vaccinating their children with rotavirus vaccine than at the beginning of the study. Subjects in intervention group 2 were more confident that they were able to afford rotavirus vaccine at the end of the study.

## Discussion

This multiple-component intervention package was effective in increasing rotavirus vaccine uptake in Hong Kong children. The intervention component targeting the removal of the financial barrier for rotavirus vaccination was the most important component to increase uptake. Considering 92%–96% vaccine effectiveness against hospitalisation,^5^ removal of the financial barrier could result in a 23%–24% reduction in incidence of rotavirus hospitalisations. Hong Kong has been achieving a stable coverage rate (more than 95%^7^) of all vaccines included in the government-funded routine CIP. Previous introduction of pneumococcal conjugate vaccine in Hong Kong showed a rapid achievement of high coverage.^7^ If this cost-saving rotavirus vaccine^6^ is incorporated in Hong Kong's universal CIP, it is likely that a similar high coverage can be anticipated given that there appear to be no negative perceptions of rotavirus vaccines within the community, leading to 57%–60% reduction in the incidence of rotavirus hospitalisation. Further study is needed to determine whether this intervention could be cost-effective if implemented in the routine public antenatal care.

Although key rotavirus information with vaccination reminders did not significantly increase uptake overall in intervention group 1 compared to the control group when we treated missing vaccination status as no vaccination, provision of key information and vaccination reminders significantly improved uptake in lower income families (Fig. 4). A previous local study showed that mothers with lower household income would have a lower perceived benefit of rotavirus vaccine and lower self-efficacy to vaccinate their children, and less likely to give rotavirus vaccine to their children.^9^ It is possible that our intervention package increased rotavirus vaccine uptake in the lower income group by increasing mothers’ perception of benefit and their self-efficacy.

The increase in rotavirus vaccine uptake in families with monthly household income HKD 30,000–39,999 was the smallest among all income groups. Fig. 3 shows that the uptake in these families were higher than those in families with household income HKD 40,000–49,999 in both control group and intervention group 1, and does not show an increasing slope as for intervention group 2 in Fig. 3. Although not statistically significant, there were more control-mothers in this income group (62%) had a plan to vaccinate their children during the immediate postpartum period than mothers in intervention groups 1 (48%) and 2 (44%). Therefore, the impact by the interventions may be less significant in this income group.

To the best of our knowledge, there have been no intervention studies or randomised controlled trials targeted to increase uptake of rotavirus vaccine alone in children, but some studies investigated interventions to increase uptakes of all childhood vaccines included in national immunisation programmes, in which rotavirus vaccine may be included. A review on interventions to increase paediatric vaccine uptakes showed that targeted and tailored interventions to parents such as reminders and education were effective in increasing paediatric vaccine uptake.^17^ Parent education included conversations with healthcare providers, posters and other informational “advertisements” in waiting or exam rooms in health facilities, or traditional educational materials such as information sheets, brochures or webpages. In addition, a local randomised controlled trial showed a multiple-component intervention package (including key influenza information with vaccination reminders) targeting postpartum mothers improved influenza vaccine uptake in Hong Kong children at two years old by 25 percent-points.^13^

This study has some limitations. First, participants were recruited only from two public hospitals but not from private hospitals. Mothers giving birth at private hospitals likely have higher household incomes and may be exposed to more rotavirus vaccination information. However, the majority (63%) of Hong Kong births were in public hospitals in 2021^18^ and the two study hospitals have a catchment of approximately 20% of all Hong Kong births. From the Hong Kong Population Census in 2021, 58.3% women aged 20–49 attained education higher than senior secondary level and the median monthly household income of families with 3 and 4 persons were HKD 34,500 and HKD 45,290 respectively.^19^ In this study, 58.9% of the subjects (aged between 18 and 47 years) attained education higher than senior secondary level, and the median monthly household income should fall in the HKD 30,000–49,999 range (23% and 18% of the subjects had monthly household income HKD 30,000–39,999 and HKD 40,000–49,999 respectively). Second, the potential differences in the background of mothers who refused and agreed to participate in the study may influence the representativeness of the study sample. Mothers who refused to take part were older and had attained a lower educational level, and fewer (56% vs 68%) of them had heard of rotavirus vaccine during the immediate postpartum period, compared to the recruited mothers. Third, there were more missing rotavirus vaccination status information in the control group and intervention group 1 than in intervention group 2. We have considered no vaccination for any missing status in the analyses, and additionally performed the main analysis on the effectiveness of the intervention for those with vaccination status only. Fourth, 33% of control-subjects agreed to the question that their vaccination decision had been influenced by the study. The 48% uptake in the control group is also higher than the best available rotavirus vaccination coverage rate in Hong Kong (33%).^7^ Participation in the study, without intervention, may already affect their vaccination decision. This may also be due to Hawthorne effect.^20^ Fifth, a few (6.7%) of the intervention-subjects could not be contacted or engaged to explain the intervention. Those mothers may not read the intervention. By intention-to-treat analysis, all intervention-subjects were considered to have obtained the intervention to provide a conservative result.

In conclusion, the intervention package consisting of a concise information sheet (containing (i) key information about the risks of rotavirus to children and the benefits of rotavirus vaccination; (ii) eligible age to receive rotavirus vaccine; (iii) a hyperlink to a government webpage showing clinics in the private sector that provide rotavirus vaccines; and (iv) guidance on searching these clinics), a vaccination reminder and tokens for free rotavirus vaccination effectively increased rotavirus vaccine uptake by 1.7 times or 33 percent-points (from 48% to 81%), compared to publicly available standard rotavirus information. The intervention package improved mothers’ confidence to make the best decision about vaccinating their children with rotavirus vaccine. The intervention package was most impactful on low-income families, emphasising the importance of removing financial barriers to vaccination to promote equity. Incorporating rotavirus vaccine into the routine CIP could further protect more young children from rotavirus infection and improve equity.

## Contributors

KHTY and EASN conceptualised and designed the study. KHTY and CCWY designed the data analysis plan, analysed and interpreted the data, have directly accessed and verified the underlying data reported in the manuscript. KHTY coordinated the study and drafted the initial manuscript. KHTY, WHT, KSL, GPGF and EASN oversight of the recruitment at the two study hospitals. All authors critically reviewed and approved the final manuscript, and confirm that they had full access to all the data in the study and accept responsibility to submit for publication.

## Data sharing statement

The data and trial protocol are accessible to researchers upon reasonable request for data sharing to the corresponding authors.

## Editor note

The Lancet Group takes a neutral position with respect to territorial claims in published maps and institutional affiliations.

## Declaration of interests

EASN received travel and accommodation support by meeting organizers to conferences and meetings (MasterClass in Vaccinology in Taipei, Taiwan in November 2023; and 9th Asian Vaccine Conference in Cebu, Philippines in November 2023), and honorarium was paid to The Chinese University of Hong Kong for participation in the MSD organised Asia Pacific Pediatrics Vaccines Regional Group Input Meeting in July 2023.

## Appendix A Supplementary data

IRVU_manuscript_appendices

IRVU Protocol NTEC
