# Supplementary material for: Multiple-component interventions to increase rotavirus vaccine uptake in children: a randomised controlled trial
Source: Lancet Reg Health West Pac. 2024 Aug 5;50:101153. doi: 10.1016/j.lanwpc.2024.101153 (PMC11357879; doi:10.1016/j.lanwpc.2024.101153)
Supplement: IRVU_manuscript_appendices [file mmc1.pdf]

Appendices of “Multiple-component interventions to increase rotavirus vaccine uptake in children: a randomised controlled trial”

Authors: Karene Hoi Ting YEUNG, Christy Ching Wun YEUNG, Wing Hung TAM, King Shun LIU, Genevieve Po Gee FUNG, E. Anthony S. NELSON

DOI: 10.1016/j.lanwpc.2024.101153

Appendix 1. Standard information from the Centre for Health Protection about rotavirus infection

香港特別行政區政府
衞生署 衞生防護中心

健康資訊

主頁 > 健康資訊 > 傳染病 > 輪狀病毒感染

輪狀病毒感染

2019年7月5日

按此瀏覽病毒性腸胃炎專題網頁

病原體

在電子顯微鏡下呈輪狀的輪狀病毒，是全球引致兒童腹瀉的最常見病因之一。輪狀病毒感染較常見於天氣較涼的季節。

病徵

主要病徵包括發燒、嘔吐和水狀腹瀉，情況一般會持續 3 - 7 天。腹痛亦是常見病徵。健康人士患上輪狀病毒腸胃炎後一般會自行痊癒，但幼童偶然會出現嚴重脫水的情況。患者在病癒後不會產生終生免疫，但再次受感染時病情則會較為輕微。

傳播途徑

主要透過口糞途徑傳播，亦可透過飲用或進食受污染的食水或食物，或接觸受污染的物件表面傳播。日間托兒中心等院舍環境較易出現輪狀病毒感染爆發。

潛伏期

約為 24 - 72小時。

治理方法

現時沒有抗病毒藥物治療輪狀病毒感染。患者應飲用充足的飲料以避免出現脫水。如懷疑出現脫水，應盡快求醫接受適當治療。

預防方法

預防輪狀病毒感染的方法跟預防其他病毒性腸胃炎相似。保持良好的個人、食物及環境衞生最為重要。

1. 保持良好的個人衞生

- 經常保持雙手清潔，尤其在處理食物或進食前，以及如廁後。洗手時應以梘液和清水清潔雙手，搓手最少20秒，用水過清並用抹手紙或乾手機弄乾。如沒有洗手設施，或雙手沒有明顯污垢時，使用含70至80%的酒精搓手液潔淨雙手亦為有效方法。
- 清理或處理嘔吐物及糞便時，須戴上手套及外科口罩，事後必須徹底洗手。
- 用膳時要使用公筷和公匙，不要與他人共享同一食物或飲料。
- 如出現嘔吐或腹瀉等腸胃病徵的人士，不應上班或上學及應向醫生求診。
- 患者或帶菌者切勿處理食物和照顧兒童、長者和缺乏免疫力的人士。

2. 保持良好的食物衞生

- 處理食物時應遵從食物安全五要點，即精明選擇（選擇安全的原材料）、保持清潔（保持雙手及用具清潔）、生熟分開（分開生熟食物）、煮熟食物（徹底煮熟食物）及安全溫度（把食物存放於安全溫度），藉以預防由食物傳播的疾病。
- 只飲用煮沸後的自來食水或出品自可靠商戶的樽裝飲品。
- 避免飲用來歷不明的冰塊所調製的飲料。
- 從衞生及可靠的地方購買新鮮食物，不要光顧無牌小販。
- 一般而言，可進食經徹底洗淨的水果皮。不過，如果希望減低患上疾病的風險，尤其是身處外地時，進食生的水果前應去掉外皮。
- 所有食物應該徹底煮熟才食用。
- 孕婦、嬰兒、幼兒、老人和免疫系統較弱等易受感染的群組，應避免進食沒有烹煮的食物（例如貝類海產，特別是生蠔）或食物內含有不會被煮熟的原料 （例如預製或預先包裝的沙律）。

*請瀏覽食物安全中心網頁，以獲得更多有關食物安全的資訊。

3. 保持良好的環境衞生

- 經常清潔和消毒常接觸的表面，如傢俬、玩具和共用物件。使用 1 比 99 稀釋家用漂白水（即把1 份 5.25% 漂白水與 99 份清水混和）消毒，待 15 － 30 分鐘後，用水清洗並抹乾。金屬表面則可用70%火酒清潔消毒。
- 用吸水力強的即棄抹巾清理可見的污物，如呼吸道分泌物、嘔吐物或排泄物，然後用1 比 49 稀釋家用漂白水（即把 1 份 5.25% 漂白水與 49 份清水混和）消毒被污染的地方及鄰近各處，待15 - 30分鐘後，用水清洗並抹乾。金屬表面則可用70%火酒清潔消毒。
- 保持室內空氣流通。
- 保持衞生設施和排水系統正常運作。
- 應妥善清潔及消毒患者曾使用的廁所和受排泄物污染的地方。

4. 接種疫苗

- 供嬰兒使用的口服輪狀病毒疫苗能有效預防此感染。家長可向家庭醫生查詢詳情。

網上資源

相關連結

美國疾病控制及預防中心（只備英文版）

世界衞生組織（只備簡體中文版）

Centre for Health Protection
Department of Health
The Government of the Hong Kong Special Administrative Region

Health Topics

Rotavirus Infection

27 December 2018

Causative agent

Rotavirus, which has a characteristic wheel-like appearance under electron microscopy, is one of the most common causes of diarrhoea among children worldwide. The disease is more commonly seen in cooler months.

Clinical features

The disease is characterised by fever, vomiting and watery diarrhoea for 3 – 7 days. Abdominal pain is also frequently reported. Rotavirus gastroenteritis is a self-limiting illness in healthy persons. However, it is occasionally associated with severe dehydration in young children. Immunity after infection is incomplete, but re-infections tend to be less severe than the original infection.

Mode of transmission

The primary mode of transmission is predominantly faecal-oral. Transmission can also occur through ingestion of contaminated water or food, or contact with contaminated surfaces. Outbreaks can occur among children in day care settings.

Incubation period

Approximately 24 – 72 hours.

Management

There is no antiviral drug to treat rotavirus infection. Drink plenty of liquids to protect against dehydration. Patients suspected to have dehydration should seek medical advice for proper management.

Prevention

Prevention of rotavirus infection is similar to that of other viral gastroenteritis. Good personal, food and environmental hygiene are the mainstay of prevention

1. Maintain good personal hygiene

- Perform hand hygiene frequently, especially before handling food or eating, and after using the toilet. Wash hands with soap and water for at least 20 seconds, then dry with a disposable paper towel or hand dryer. If hand washing facilities are not available, or when hands are not visibly soiled, hand hygiene with 70-80% alcohol-based handrub is an effective alternative.
- Wear gloves and a surgical mask while disposing of or handling vomitus and faeces, and wash hands thoroughly afterwards
- Use serving chopsticks and spoons at meal time. Do not share food and drinks with others.
- Refrain from work or attending class at school, and seek medical advice if suffering from vomiting or diarrhoea.

- Exclude infected persons and asymptomatic carriers from handling food and from providing care to children, the elderly and immunocompromised people.

2. Maintain good food hygiene

- Adopt the 5 Keys to Food Safety in handling food, i.e. Choose (Choose safe raw materials); Clean (Keep hands and utensils clean); Separate (Separate raw and cooked food); Cook (Cook thoroughly); and Safe Temperature (Keep food at safe temperature) to prevent foodborne diseases.
- Drink only boiled water from the mains or bottled drinks from reliable sources.
- Avoid drinks with ice of unknown origin.
- Purchase fresh food from hygienic and reliable sources. Do not patronise illegal hawkers.
- In general, you can eat fruit skin after thorough washing. However, if you would like to reduce the risk of illness especially when you travel abroad, peel raw fruit before you eat and do not eat the peelings.
- Cook all food thoroughly before consumption.
- Susceptible populations (e.g. pregnant women, infants, young children, the elderly and people with weakened immune systems) should avoid eating foods that are consumed without heat treatment (e.g. shellfish, especially oyster, to be consumed raw) or foods containing ingredients that are not cooked (e.g. pre-prepared or pre-packaged salads).

* Please visit the website of Centre for Food Safety for more information on food safety.

3. Maintain good environmental hygiene

- Regularly clean and disinfect frequently touched surfaces such as furniture, toys and commonly shared items with 1:99 diluted household bleach (mixing 1 part of 5.25% bleach with 99 parts of water), leave for 15-30 minutes, and then rinse with water and keep dry. For metallic surface, disinfect with 70% alcohol.
- Use absorbent disposable towels to wipe away obvious contaminant such as respiratory secretions, and then disinfect the surface and neighbouring areas with 1:49 diluted household bleach (mixing 1 part of 5.25% bleach with 49 parts of water), leave for 15-30 minutes and then rinse with water and keep dry. For metallic surface, disinfect with 70% alcohol.
- Maintain good indoor ventilation.
- Maintain proper sanitary facilities and drainage system.
- Clean and disinfect toilets used by infected person and the soiled areas.

4. Vaccination

- There are oral vaccines for infants which can prevent rotavirus infection effectively. Parents can approach their family doctors for further advice and information.

For details, please refer to the thematic webpage of Viral Gastroenteritis.

e-Resources

Related Links

Centers for Disease Control and Prevention, USA

World Health Organization

Appendix 2. Information sheet provided to subjects in the intervention group 1

IRVU G1

7 只選擇「輪狀病毒疫苗」，取消所有其他選項

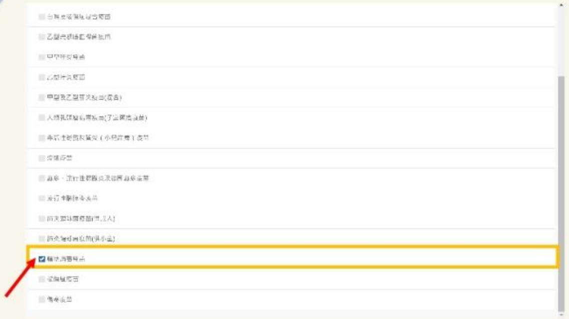

8 向上滑，在右上角按「完成」

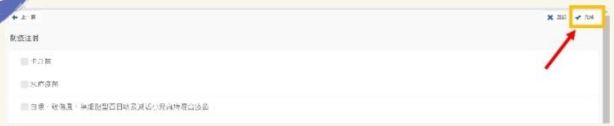

9 向下滑，確保在選項「所提供的服務」嘅右邊有數字「1」，然後按「搜尋」，就能夠查到所選擇地區有提供輪狀病毒疫苗嘅私家診所。

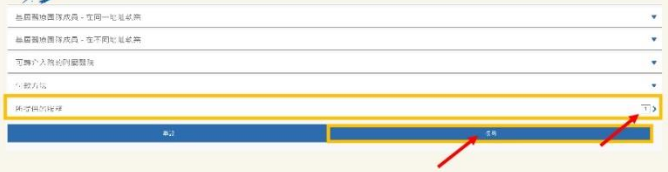

保護你嘅孩子
食口服輪狀病毒疫苗

輪狀病毒係咩嚟？

輪狀病毒會引致腹瀉和嘔吐

邊個會感染？

所有 5 歲以下嘅小朋友都會感染過輪狀病毒

輪狀病毒嚴唔嚴重？

喺香港，大約每 30 個 5 歲以下嘅小朋友，就會有 1 個因感染輪狀病毒而入院

有部份小朋友可能因感染輪狀病毒而抽搐

如小朋友喺感染輪狀病毒後出現嚴重腹瀉和嘔吐，又未能得到適當治療，就有可能死亡

輪狀病毒疫苗係咩嚟？效用高唔高？

現時香港有兩種輪狀病毒疫苗，都係口服嘅，唔係注射嘅針

輪狀病毒疫苗有 90%效能預防 5 歲以下嘅香港小朋友感染輪狀病毒

係咪新疫苗嚟？

唔係，由 2006 年開始，已經可以喺香港接種輪狀病毒疫苗

我 BB 應該幾時接種呢個疫苗？

服食輪狀病毒疫苗嘅歲數同打白喉／破傷風混合針（DTaP-IPV）及肺炎球菌針（PCV）嘅歲數一樣。輪狀病毒疫苗可以同呢兩支針同時或之前或之後接種。

兩種輪狀病毒疫苗嘅接種時間為：

兩劑輪狀病毒疫苗：2 個月同 4 個月大

三劑輪狀病毒疫苗：2 個月，4 個月同 6 個月大

當你嘅小朋友大概 6 至 8 週大時，我哋會發送短訊提你帶小朋友去服食。

口服輪狀病毒疫苗要幾多錢？

輪狀病毒疫苗並未列入喺香港兒童免疫接種計劃入面，父母需要自費為小朋友接種疫苗，價錢因不同診所而異。

邊度有得服食輪狀病毒疫苗？

部份私家診所會 **收費** 為小朋友提供輪狀病毒疫苗，
你可以根據以下步驟搜索呢啲診所嘅聯繫方法：

1 上「基層醫療指南」網頁：https://apps.pcdirectory.gov.hk/Mobile/

2 喺「我正在找尋...」下選擇「西醫」

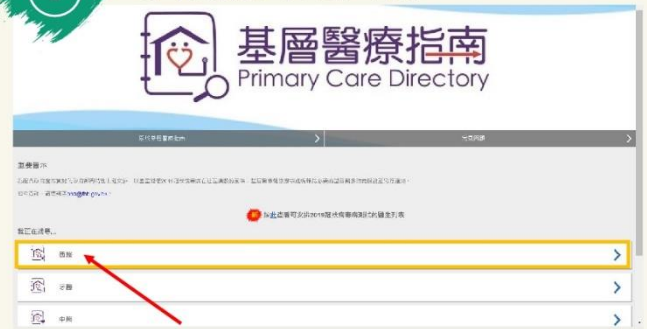

3 選擇地區

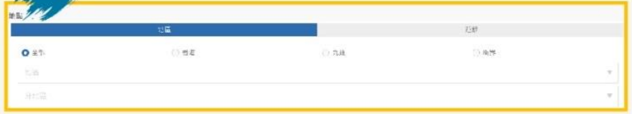

4 向下滑，喺「執業資料」下選擇「所提供的服務」

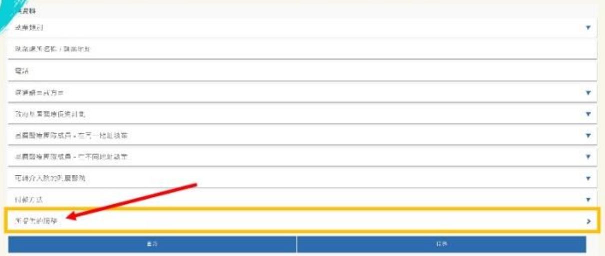

5 只選擇「疾病預防及促進健康」，然後按右邊嘅箭嘴

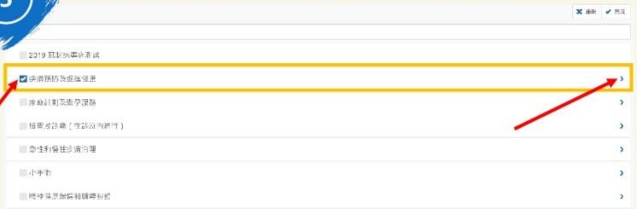

6 只選擇「防疫注射」，取消所有其他選項，然後按右邊嘅箭嘴

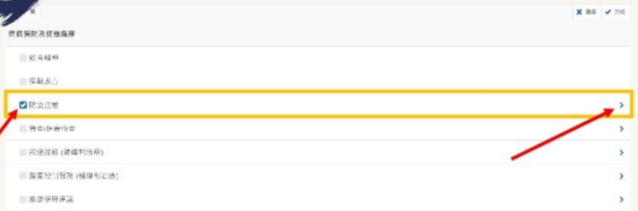

English translation:

Protect Your Child with Rotavirus Vaccine (Oral)

What is rotavirus?
Rotavirus causes diarrhoea and vomiting

Who will be infected?
All children will be infected with rotavirus by the age of 5 years

Is it serious?
In Hong Kong about 1 in 30 children will be admitted to hospital with rotavirus by the age of 5 years
Some children can get convulsions from rotavirus infection
Children with severe vomiting and diarrhoea from rotavirus infection who do not get treatment can die

What is rotavirus vaccine and how effective is it?
There are two rotavirus vaccines available in Hong Kong, both are given by mouth –not an injection
Rotavirus vaccines are at least 90% effective in Hong Kong children below the age of 5 years

Is it a new vaccine?
No, rotavirus vaccines have been available in Hong Kong since 2006

When should my child get the vaccine?
Rotavirus vaccine is given at the same age as DTaP-IPV and PCV vaccines
Rotavirus vaccine can be given before, after or at the same time as these other vaccines
The timing to receive the two types of rotavirus vaccines are:

2-dose rotavirus vaccine: 2 months (6-10 weeks) and 4 months
3-dose rotavirus vaccine: 2 months (6-10 weeks), 4 months and 6 months

We will send a text message reminder for vaccination when your child is about 6-8 weeks old

How much does the vaccine cost?

Rotavirus vaccine has yet to be included in the Childhood Immunisation Programme. Parents have to pay out of pocket but the price varies at different health facilities.

Where to get the vaccine?

There are a number of private clinics providing rotavirus vaccine to children **<u>WITH CHARGE FOR VACCINE</u>**. You may follow the steps below to search for the contact details of these clinics.

1. Go to the 'Primary Care Directory' webpage: https://apps.pcdirectory.gov.hk/Mobile/
2. Click 'Doctors' under 'I am looking for…'

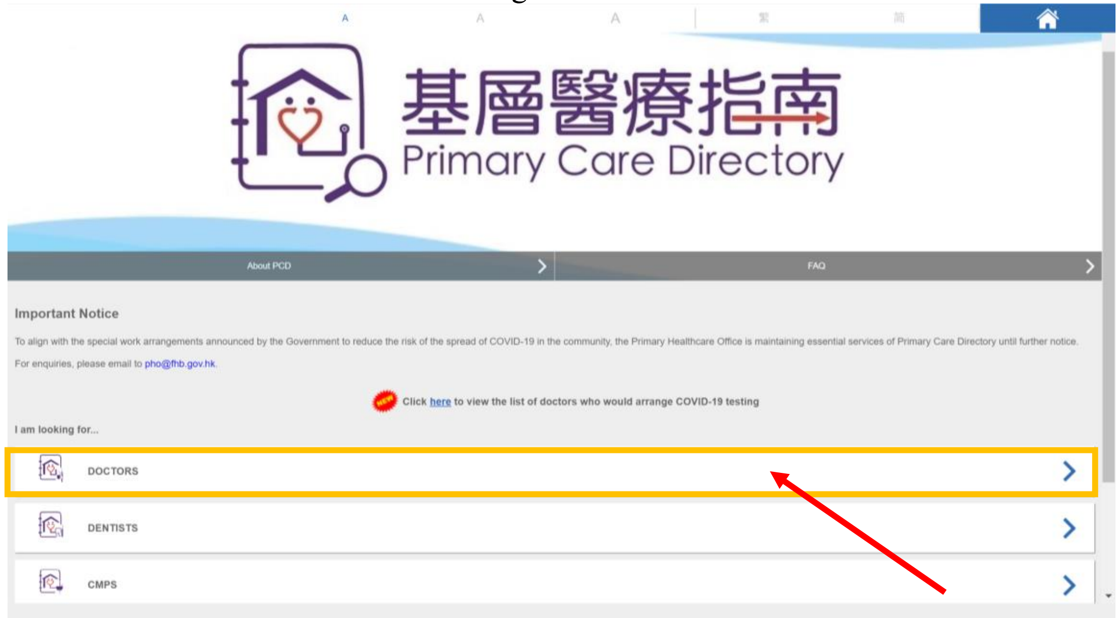

3. Choose location

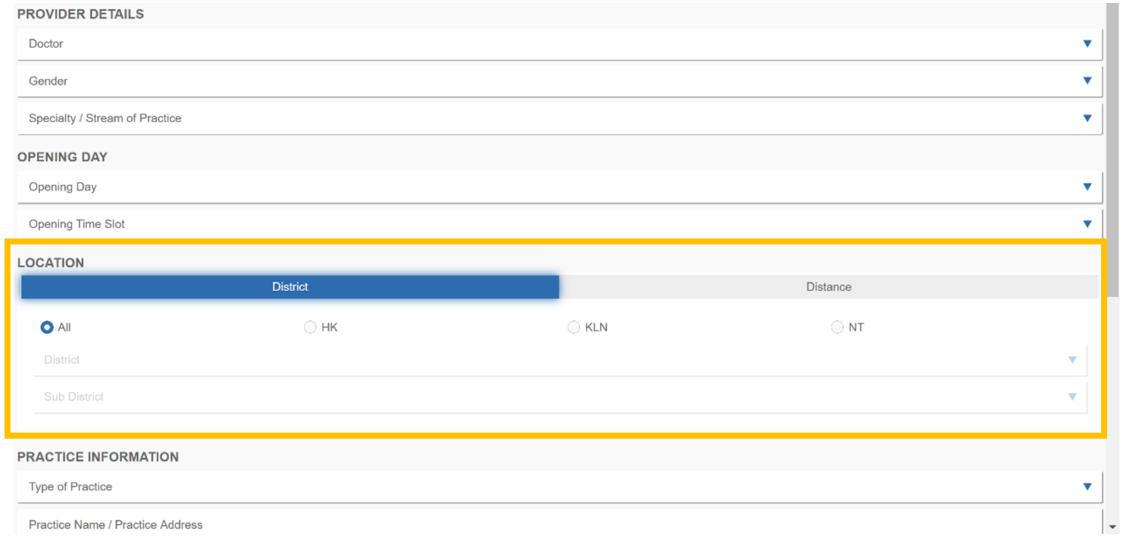

4. Scroll down and choose ‘Services Provision’ under the ‘Practice Information’ at the bottom

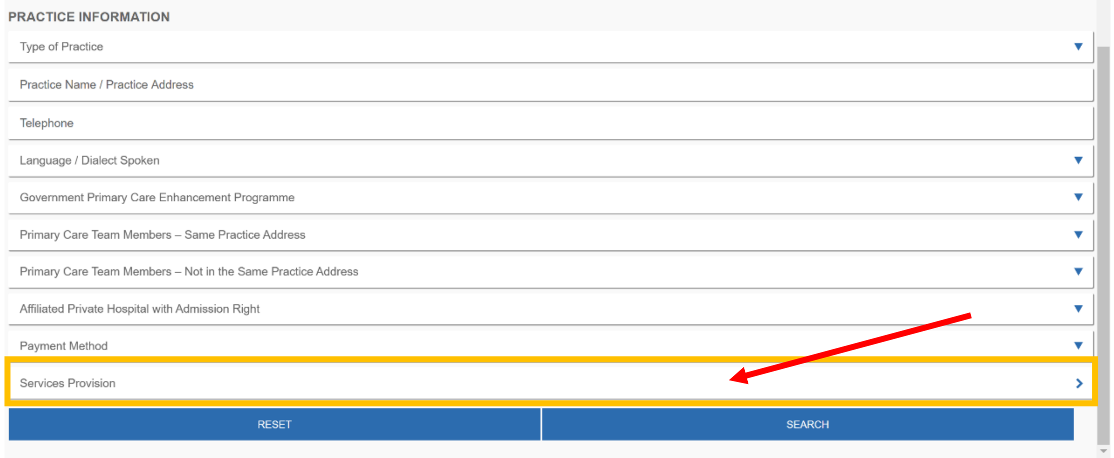

5. Click ‘Disease Prevention and Health Promotion’ and press the arrow on the right

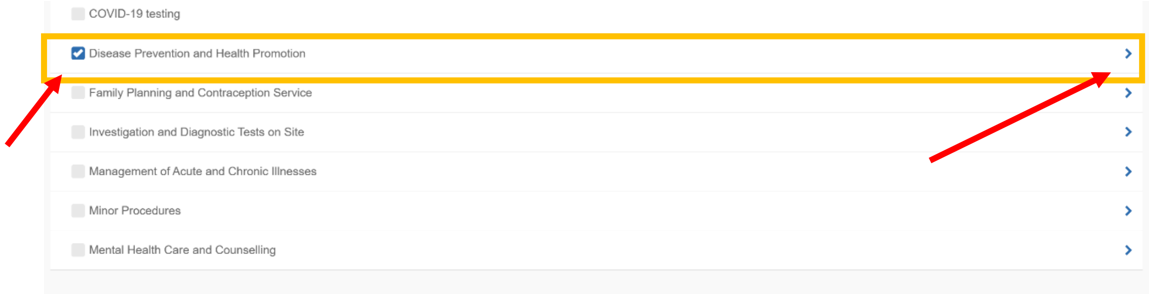

6. Deselect all choices under ‘Disease Prevention and Health Promotion’ except ‘Immunisation’. Then press the arrow on the right.

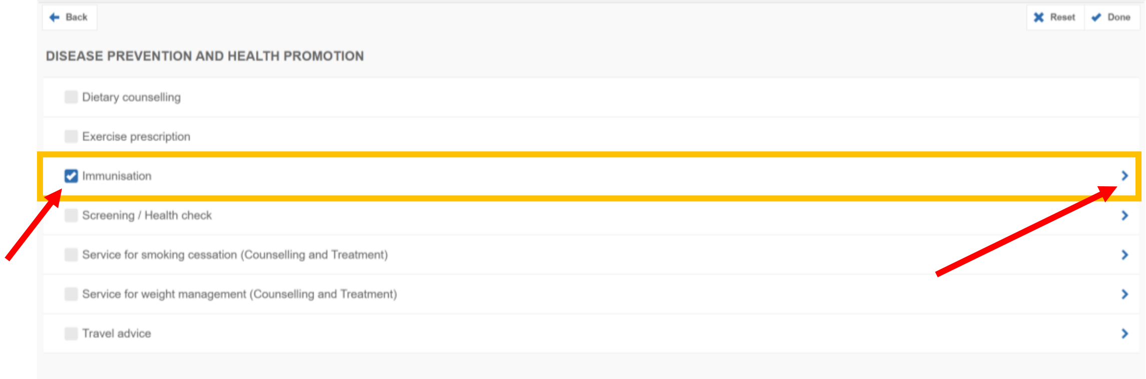

7. Deselect all choices under ‘Immunisation’ except ‘Rotavirus vaccine’

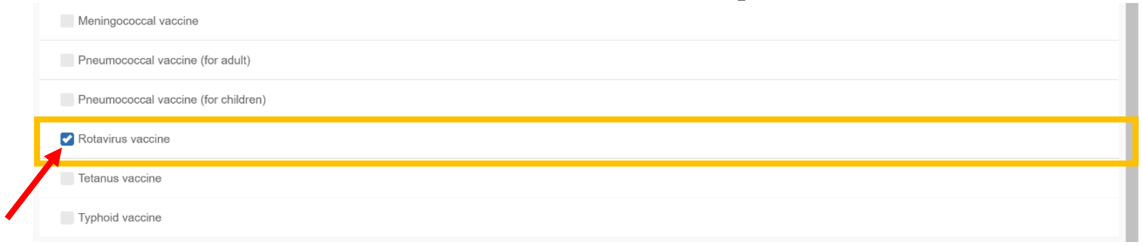

8. Scroll up and press ‘done’ at the top right corner

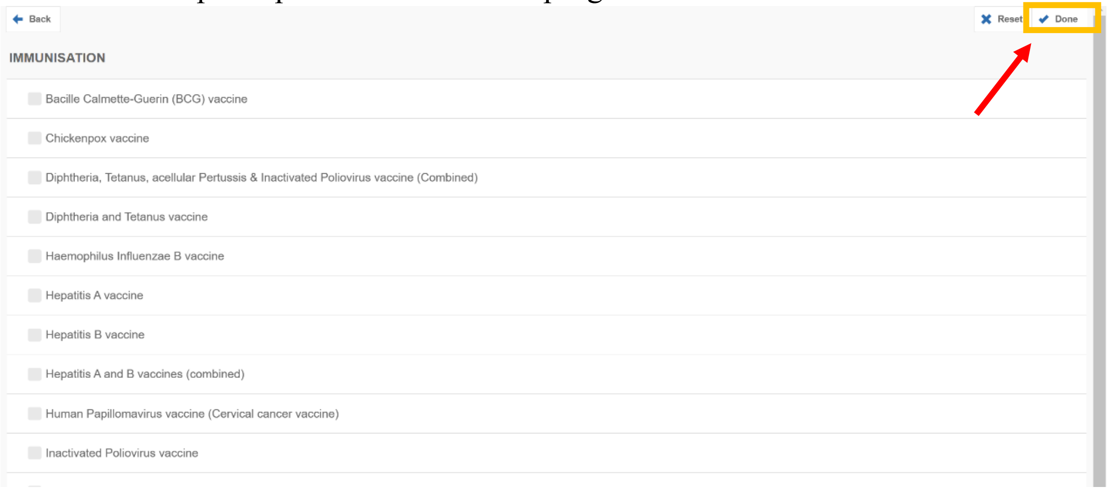

9. Scroll down. Make sure there is a number ‘1’ on the right end of the option ‘Service Provision’. Then press ‘SEARCH’. Finally, you can see a list of private clinics providing rotavirus vaccines in the district you selected.

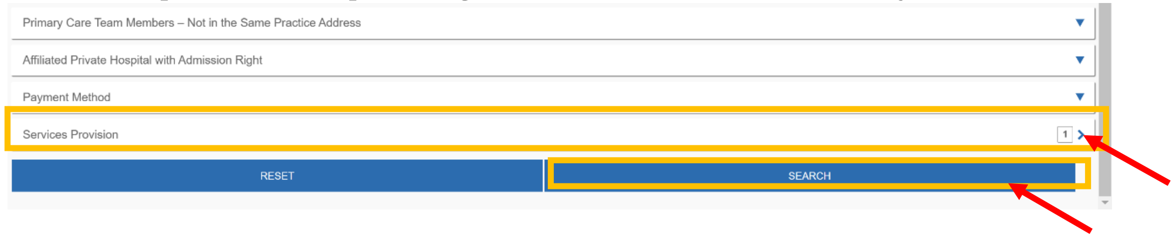

Appendix 3. Information sheet provided to subjects in the intervention group 2
(a) For a clinic within the United Christian Nethersole Community Health Service located in Kwun Tong

IRVU G2 – Kwun Tong

7 只選擇「輪狀病毒疫苗」，取消所有其他選項

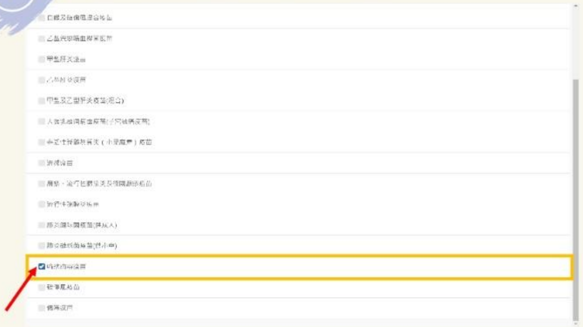

8 向上滑，在右上角按「完成」

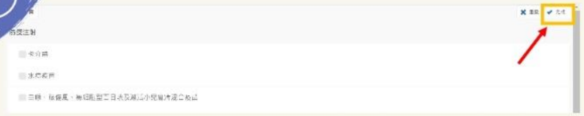

9 向下滑，確保在選項「所提供的服務」嘅右邊有數字「1」，然後按「搜尋」，就能夠查到所選擇地區有提供輪狀病毒疫苗嘅私家診所。

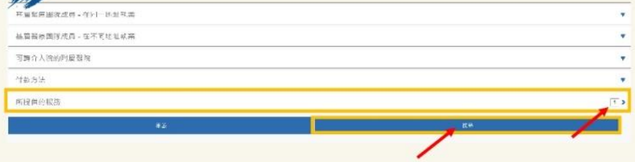

保護你嘅孩子
食口服輪狀病毒疫苗

輪狀病毒係咩嚟？
輪狀病毒會引致腹瀉和嘔吐

邊個會感染？
所有 5 歲以下嘅小朋友都會感染過輪狀病毒

輪狀病毒嚴唔嚴重？
喺香港，大約每 30 個 5 歲以下嘅小朋友，就會有 1 個因感染輪狀病毒而入院
有部份小朋友可能因感染輪狀病毒而抽搐
如小朋友喺感染輪狀病毒後出現嚴重腹瀉和嘔吐，又未能得到適當治療，就有可能死亡

輪狀病毒疫苗係咩嚟？效用高唔高？
現時香港有兩種輪狀病毒疫苗，都係口服嘅，唔係注射嘅針
輪狀病毒疫苗有 90%效能預防 5 歲以下嘅香港小朋友感染輪狀病毒

係咪新疫苗嚟？
唔係，由 2006 年開始，已經可以喺香港接種輪狀病毒疫苗

我 BB 應該幾時接種呢個疫苗？
服食輪狀病毒疫苗嘅歲數同打白喉／破傷風混合針（DTaP-IPV）及肺炎球菌針（PCV）嘅歲數一樣。輪狀病毒疫苗可以同呢兩支針同時或之前或之後接種 。

兩種輪狀病毒疫苗嘅接種時間為：

兩劑輪狀病毒疫苗：2 個月同 4 個月大

三劑輪狀病毒疫苗：2 個月，4 個月同 6 個月大

當你嘅小朋友大概 6 至 8 週大時，我哋會發送短訊提你帶小朋友去服食。

口服輪狀病毒疫苗要幾多錢？
輪狀病毒疫苗並未列入喺香港兒童免疫接種計劃入面，父母需要自費為小朋友接種疫苗，價錢因不同診所而異。

作為呢項研究嘅一部份，只要到以下嘅醫療中心出示附上嘅代用券，你嘅小朋友就可以**免費**服食兩劑輪狀病毒疫苗。第一劑會喺大概 6 至 10 週大時服食（**必須 20 週前**）；而第二劑就要喺服食**第一劑後至少 4 星期，同 24 週大前服食**。

邊度有得 **免費** 服食輪狀病毒疫苗？

賽馬會和樂社區健康中心 (需預約)

地址：
觀塘協和街和樂邨居安樓 26 - 33 號地下

開放時間:
一、三 、五: 8:30am – 1pm; 2pm – 5:30pm
二、四: 8:30am – 1pm; 2pm – 7pm
六: 8:30am – 1pm; 2pm – 5pm

電話: 2344 3444
WhatsApp: 5625 3918
(回覆時段: 一至五: 9am - 5pm; 六: 9am - 1pm)

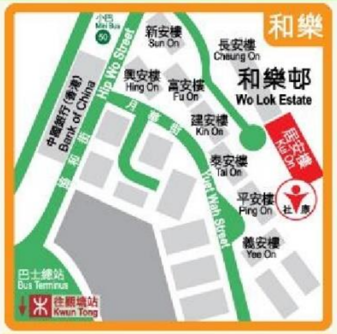

交通：港鐵觀塘站 A1 出口 →
綠色小巴 50 號 (往裕民坊方向)
→ 和樂邨

搜索其他提供 **自費** 輪狀病毒疫苗嘅私家診所

你可以根據以下步驟搜索呢啲診所嘅聯繫方法：

1. 上「基層醫療指南」網頁：https://apps.pcdirectory.gov.hk/Mobile/

2. 喺「我正在找尋...」下選擇「西醫」

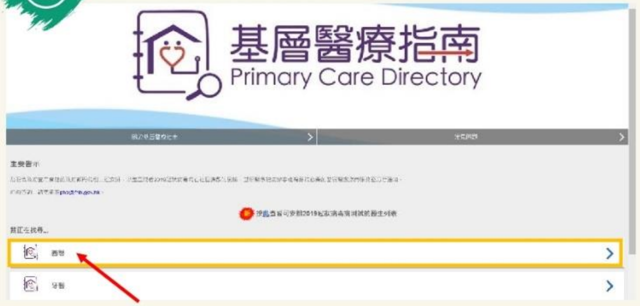

3. 選擇地區

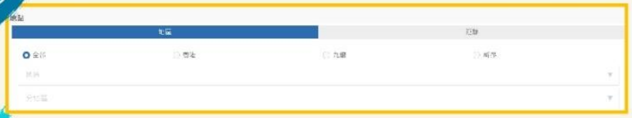

4. 向下滑，喺「執業資料」下選擇「所提供的服務」

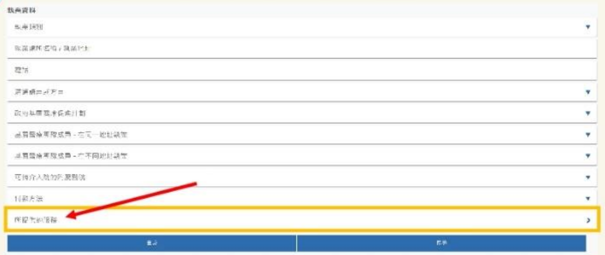

5. 只選擇「疾病預防及促進健康」，然後按右邊嘅箭嘴

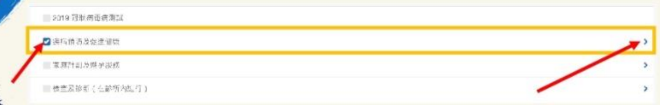

6. 只選擇「防疫注射」，取消所有其他選項，然後按右邊嘅箭嘴

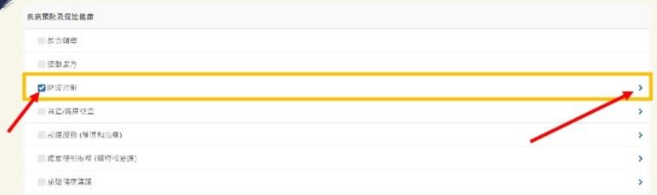

(b) For a clinic within the United Christian Nethersole Community Health Service located in Tai Po

IRVU G2 – Tai Po

7 只選擇「輪狀病毒疫苗」，取消所有其他選項

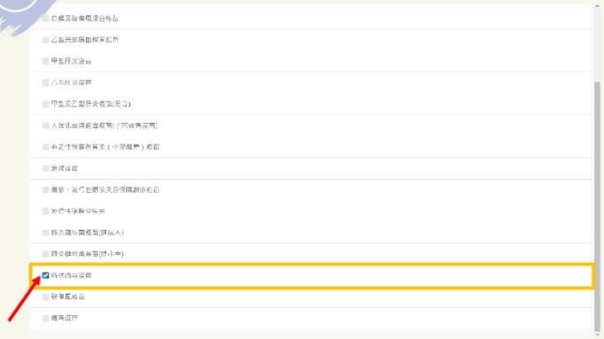

8 向上滑，在右上角按「完成」

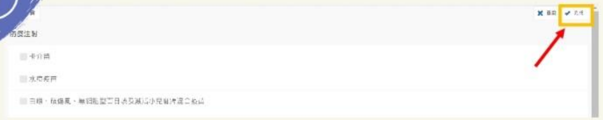

9 向下滑，確保在選項「所提供的服務」嘅右邊有數字「1」，然後按「搜尋」，就能夠查到所選擇地區有提供輪狀病毒疫苗嘅私家診所。

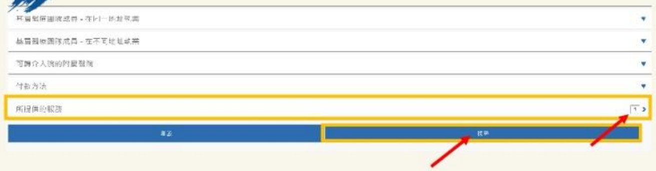

保護你嘅孩子
食口服輪狀病毒疫苗

輪狀病毒係咩嚟？
輪狀病毒會引致腹瀉和嘔吐

邊個會感染？
所有 5 歲以下嘅小朋友都會感染過輪狀病毒

輪狀病毒嚴唔嚴重？
喺香港，大約每 30 個 5 歲以下嘅小朋友，就會有 1 個因感染輪狀病毒而入院
有部份小朋友可能因感染輪狀病毒而抽搐
如小朋友喺感染輪狀病毒後出現嚴重腹瀉和嘔吐，又未能得到適當治療，就有可能死亡

輪狀病毒疫苗係咩嚟？效用高唔高？
現時香港有兩種輪狀病毒疫苗，都係口服嘅，唔係注射嘅針
輪狀病毒疫苗有 90%效能預防 5 歲以下嘅香港小朋友感染輪狀病毒

係咪新疫苗嚟？
唔係，由 2006 年開始，已經可以喺香港接種輪狀病毒疫苗

我 BB 應該幾時接種呢個疫苗？
服食輪狀病毒疫苗嘅歲數同打白喉／破傷風混合針（DTaP-IPV）及肺炎球菌針（PCV）嘅歲數一樣。輪狀病毒疫苗可以同呢兩支針同時或之前或之後接種 。

兩種輪狀病毒疫苗嘅接種時間為：
兩劑輪狀病毒疫苗：2 個月同 4 個月大
三劑輪狀病毒疫苗：2 個月，4 個月同 6 個月大
當你嘅小朋友大概 6 至 8 週大時，我哋會發送短訊提你帶小朋友去服食。

口服輪狀病毒疫苗要幾多錢？
輪狀病毒疫苗並未列入喺香港兒童免疫接種計劃入面，父母需要自費為小朋友接種疫苗，價錢因不同診所而異。

作為呢項研究嘅一部份，只要到以下嘅醫療中心出示附上嘅代用券，你嘅小朋友就可以**免費**服食兩劑輪狀病毒疫苗。第一劑會喺大概 6 至 10 週大時服食（**必須 20 週前**）；而第二劑就要喺服食**第一劑後至少 4 星期，同 24 週大前服食**。

邊度有得 免費 服食輪狀病毒疫苗？

廣福社區健康中心 (需預約)

地址：
新界大埔廣福邨廣仁樓地下 19 號

開放時間:
一至五: 8:30am - 1pm; 2:30pm - 7pm
六: 8:30am - 1pm; 2pm - 5pm

電話: 2638 3846
WhatsApp: 6226 5342
(回覆時段: 一至五: 9am - 5pm; 六: 9am - 1pm)

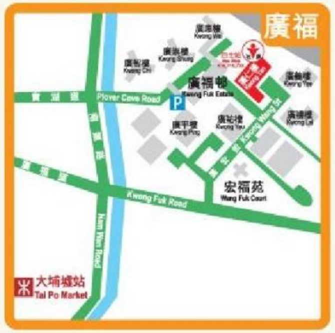

交通：港鐵大埔墟站 A3 出口 → K18 港鐵接駁巴士→ 廣福邨

搜索其他提供 自費 輪狀病毒疫苗嘅私家診所

你可以根據以下步驟搜索呢啲診所嘅聯繫方法：

1. 上「基層醫療指南」網頁：https://apps.pcdirectory.gov.hk/Mobile/

2. 喺「我正在找尋...」下選擇「西醫」

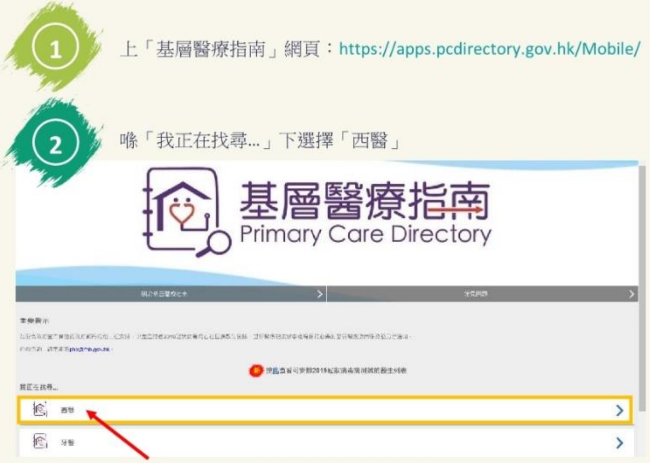

3. 選擇地區

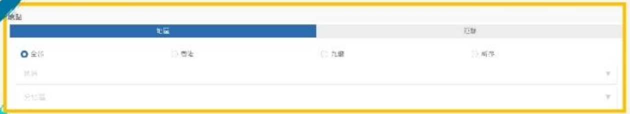

4. 向下滑，喺「執業資料」下選擇「所提供的服務」

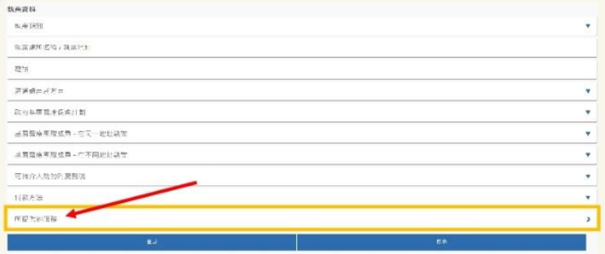

5. 只選擇「疾病預防及促進健康」，然後按右邊嘅箭嘴

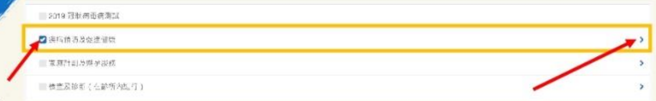

6. 只選擇「防疫注射」，取消所有其他選項，然後按右邊嘅箭嘴

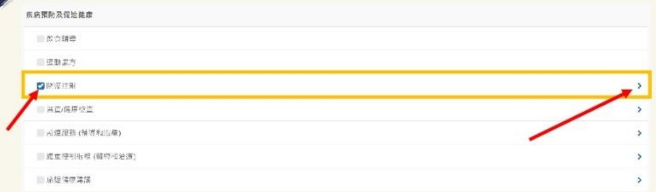

English translation:

Protect Your Child with Rotavirus Vaccine (Oral)

What is rotavirus?
Rotavirus causes diarrhoea and vomiting

Who will be infected?
All children will be infected with rotavirus by the age of 5 years

Is it serious?
In Hong Kong about 1 in 30 children will be admitted to hospital with rotavirus by the age of 5 years
Some children can get convulsions from rotavirus infection
Children with severe vomiting and diarrhoea from rotavirus infection who do not get treatment can die

What is rotavirus vaccine and how effective is it?
Rotavirus vaccine is given by mouth – it is not an injection
Rotavirus vaccines are at least 90% effective in Hong Kong children below the age of 5 years

Is it a new vaccine?
No, two rotavirus vaccines have been available in Hong Kong since 2006

When should my child get the vaccine?
Rotavirus vaccine is given at the same age as DTaP-IPV and PCV vaccines
Rotavirus vaccine can be given before, after or at the same time as these other vaccines

2-dose: 2 months and 4 months
3-dose: 2 months, 4 months and 6 months

We will send a text message reminder for vaccination when your child is about 6-8 weeks old

How much does the vaccine cost?

Rotavirus vaccine has been available in Hong Kong for use since 2006 but yet to be included in the Childhood Immunisation Programme. Parents have to pay out of pocket but the price varies at different health facilities.

As part of this study we have arranged for your child to receive a course of rotavirus vaccine (2 or 3 doses) at **<u>no cost</u>** to you or your family if you attend the following clinics with the attached token when your child is 6-10 weeks old

Where to get the vaccine?

UCN community health centre
Address:
Tel.:
Opening hours:

There are a number of private clinics providing rotavirus vaccine to children with charge. You may follow the steps below to search for the contact details of these clinics.

1. Go to the 'Primary Care Directory' webpage: https://apps.pcdirectory.gov.hk/Mobile/
2. Click 'Doctors' under 'I am looking for…'

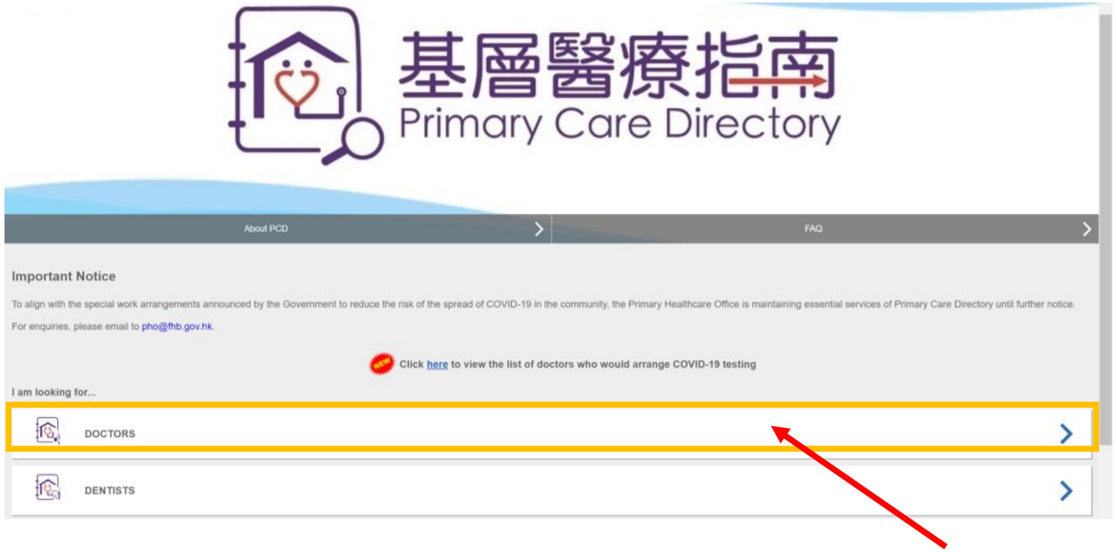

3. Choose location

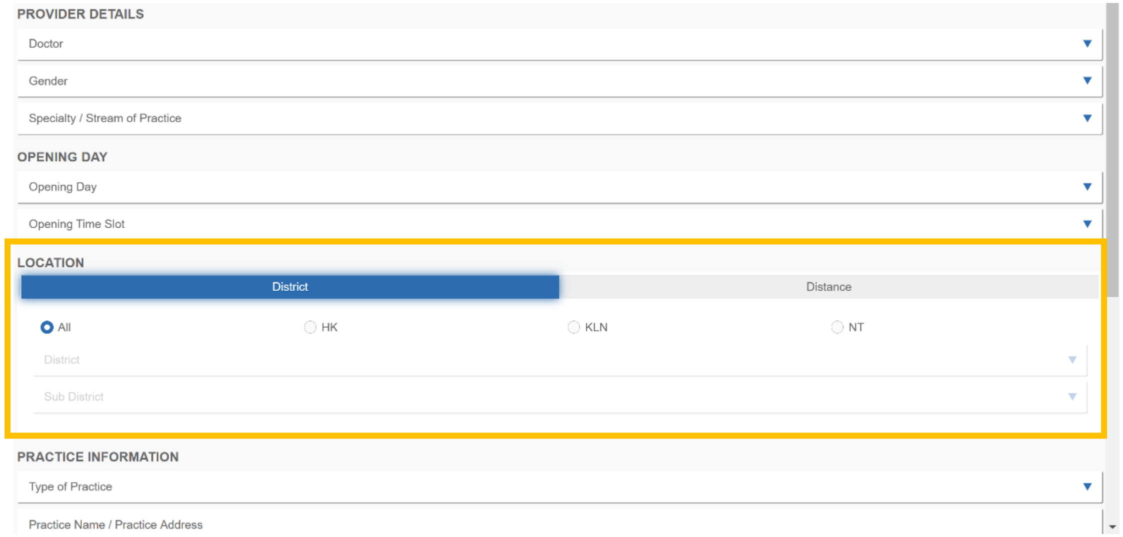

4. Scroll down and choose ‘Services Provision’ under the ‘Practice Information’ at the bottom

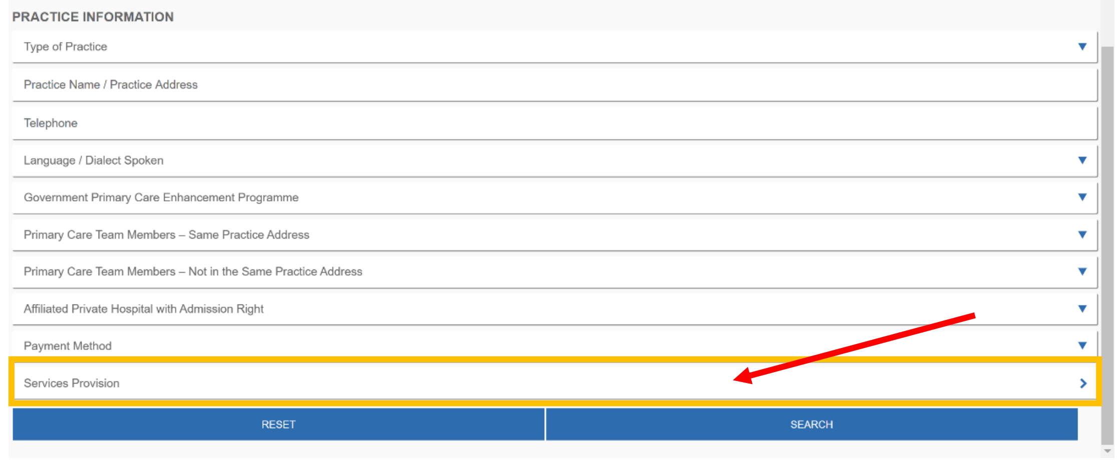

5. Click ‘Disease Prevention and Health Promotion’ and press the arrow on the right

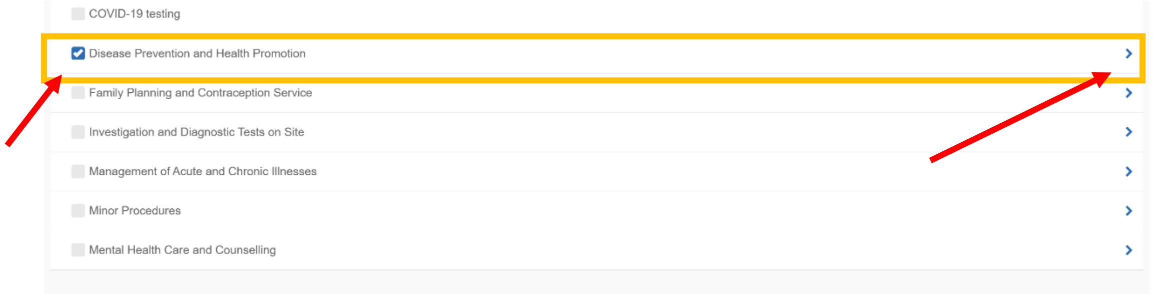

6. Deselect all choices under ‘Disease Prevention and Health Promotion’ except ‘Immunisation’. Then press the arrow on the right.

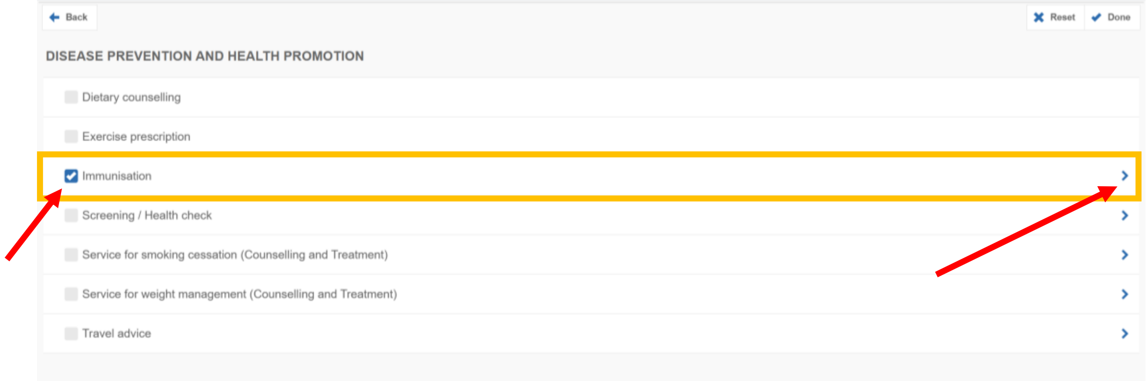

7. Deselect all choices under ‘Immunisation’ except ‘Rotavirus vaccine’

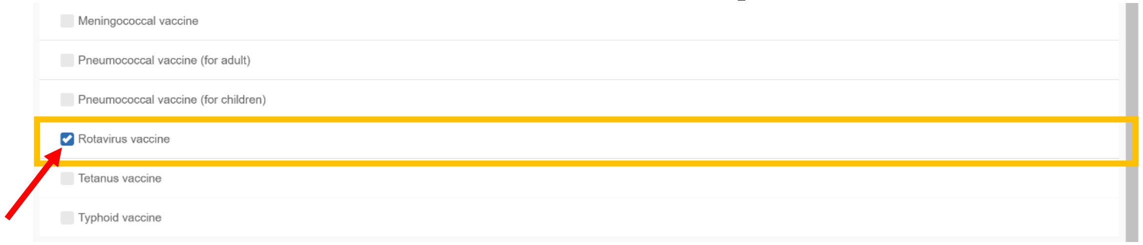

8. Scroll up and press ‘done’ at the top right corner

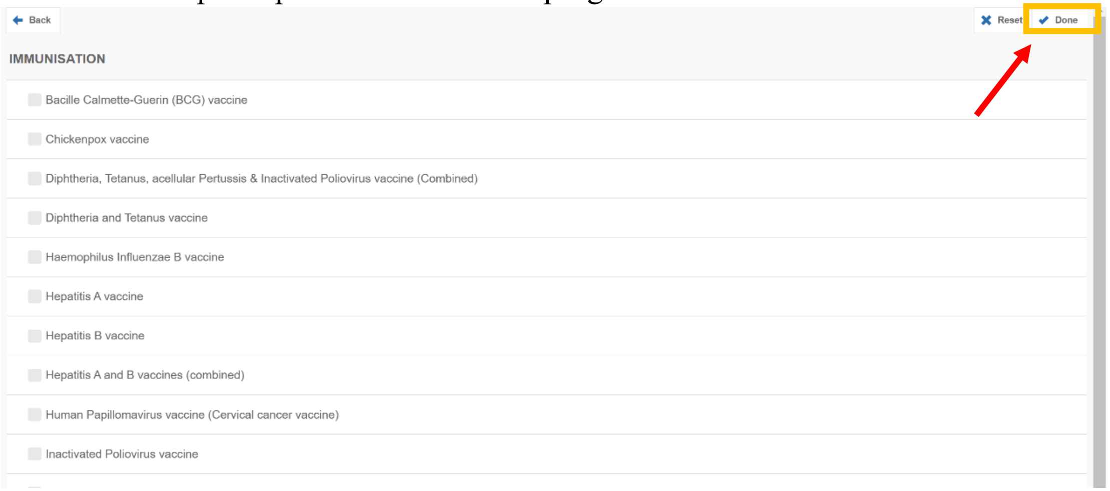

9. Scroll down. Make sure there is a number ‘1’ on the right end of the option ‘Service Provision’. Then press ‘SEARCH’. Finally, you can see a list of private clinics providing rotavirus vaccines in the district you selected.

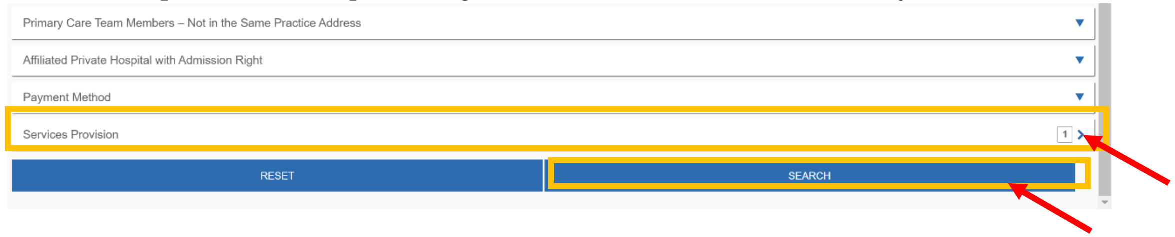

Appendix 4. Tokens provided to subjects in the intervention group 2 for free rotavirus vaccination

(a) For a clinic within the United Christian Nethersole Community Health Service located in Kwun Tong

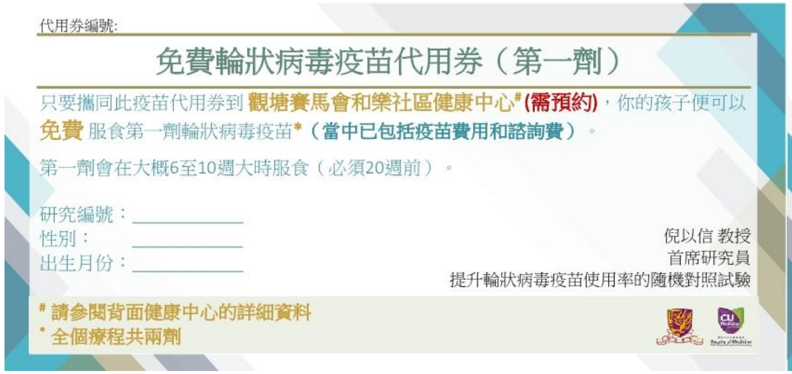

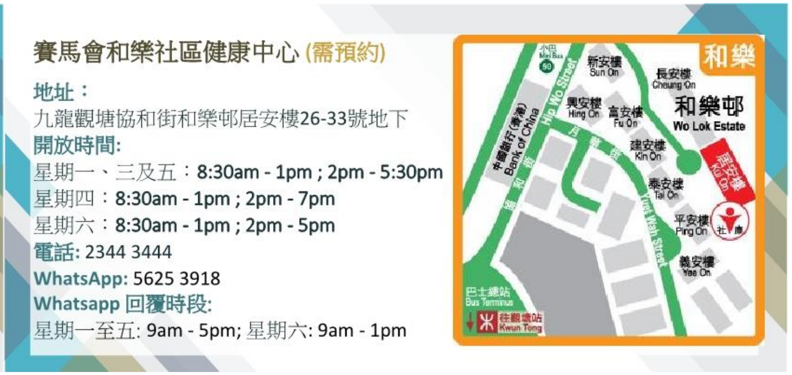

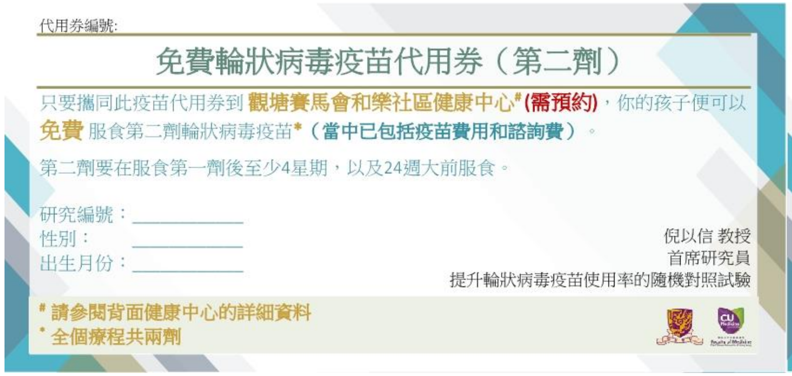

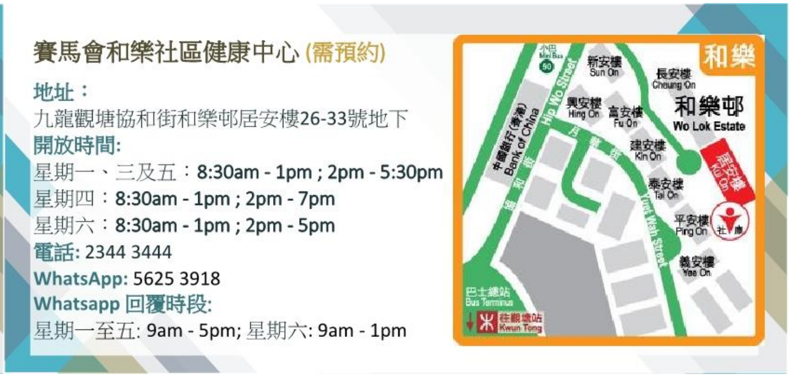

English translation:

Token no.:

Token for FREE rotavirus vaccine (first dose)

Please bring this token to the **Jockey Club Wo Lok Community Health Center# (appointment needed)**. Your child can receive the first dose of rotavirus vaccine* at **<u>no cost (including vaccine cost and consultation fee)</u>**.

The first dose needs to be received at around 6-10 weeks old (must be before 20 weeks old).

Study no.:
Gender:
Month of birth:

EAS Nelson
Principal investigator
Increasing rotavirus vaccine uptake: a randomised controlled trail

Please refer to the details of the Community Health Center at the back of the token
* The full course includes 2 doses

Jockey Club Wo Lok Community Health Center (appointment needed)

Address:	Unit 26-33, G/F, Kui On House, Wo Lok Estate, Kwun Tong, Kowloon
Opening hours:	
Mon/Wed/Fri:	8:30am - 1pm; 2pm - 5:30pm
Thu:	8:30am - 1pm; 2pm - 7pm
Sat:	8:30am - 1pm; 2pm - 5pm
Tel.:	2344 3444
WhatsApp:	5625 3918
WhatsApp responding period:	
Mon-Fri:	9am - 5pm
Sat:	9am - 1pm

Token no.:

Token for FREE rotavirus vaccine (second dose)

Please bring this token to the **Jockey Club Wo Lok Community Health Center# (appointment needed)**. Your child can receive the second dose of rotavirus vaccine* at **<u>no cost (including vaccine cost and consultation fee)</u>**.

The second dose needs to be received at least 4 weeks after the first dose, and by 24 weeks old.

Study no.:
Gender:
Month of birth:

EAS Nelson
Principal investigator
Increasing rotavirus vaccine uptake: a randomised controlled trail

Please refer to the details of the Community Health Center at the back of the token
* The full course includes 2 doses

Jockey Club Wo Lok Community Health Center (appointment needed)

Address:	Unit 26-33, G/F, Kui On House, Wo Lok Estate, Kwun Tong, Kowloon
Opening hours:	
Mon/Wed/Fri:	8:30am - 1pm; 2pm - 5:30pm
Thu:	8:30am - 1pm; 2pm - 7pm
Sat:	8:30am - 1pm; 2pm - 5pm
Tel.:	2344 3444
WhatsApp:	5625 3918
WhatsApp responding period:	
Mon-Fri:	9am - 5pm
Sat:	9am - 1pm

(b) For a clinic within the United Christian Nethersole Community Health Service located in Tai Po

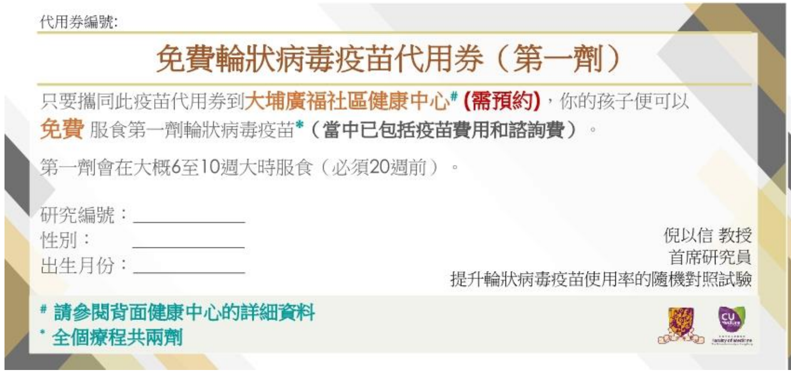

代用券編號:

免費輪狀病毒疫苗代用券（第一劑）

只要攜同此疫苗代用券到大埔廣福社區健康中心# (需預約)，你的孩子便可以**免費** 服食第一劑輪狀病毒疫苗*（當中已包括疫苗費用和諮詢費）。

第一劑會在大概6至10週大時服食（必須20週前）。

研究編號：__________

性別：__________

出生月份：__________

倪以信 教授
首席研究員
提升輪狀病毒疫苗使用率的隨機對照試驗

請參閱背面健康中心的詳細資料

* 全個療程共兩劑

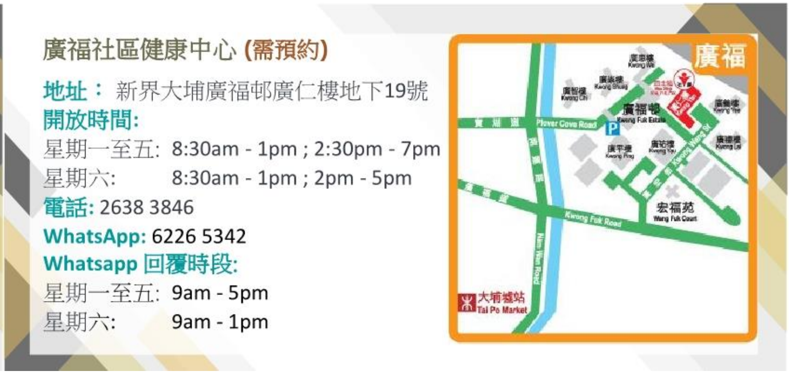

廣福社區健康中心 (需預約)

地址： 新界大埔廣福邨廣仁樓地下19號

開放時間:

星期一至五: 8:30am - 1pm ; 2:30pm - 7pm

星期六: 8:30am - 1pm ; 2pm - 5pm

電話: 2638 3846

WhatsApp: 6226 5342

Whatsapp 回覆時段:

星期一至五: 9am - 5pm

星期六: 9am - 1pm

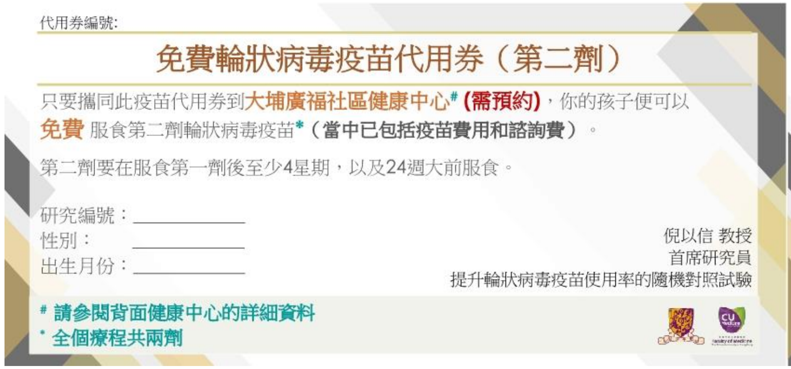

代用券編號:

免費輪狀病毒疫苗代用券（第二劑）

只要攜同此疫苗代用券到大埔廣福社區健康中心# (需預約)，你的孩子便可以**免費** 服食第二劑輪狀病毒疫苗*（當中已包括疫苗費用和諮詢費）。

第二劑要在服食第一劑後至少4星期，以及24週大前服食。

研究編號：__________

性別：__________

出生月份：__________

倪以信 教授
首席研究員
提升輪狀病毒疫苗使用率的隨機對照試驗

請參閱背面健康中心的詳細資料

* 全個療程共兩劑

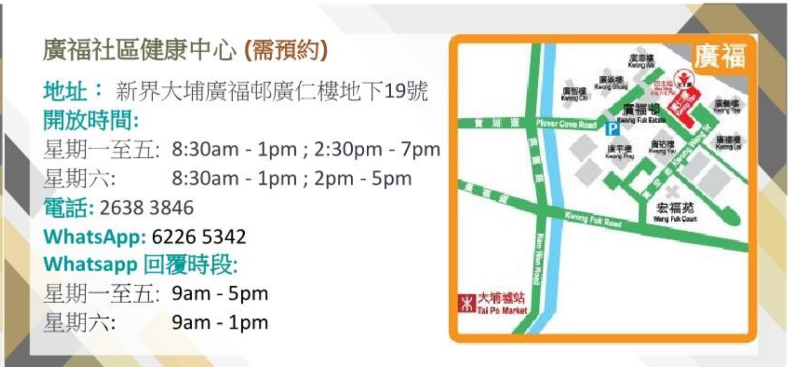

廣福社區健康中心 (需預約)

地址： 新界大埔廣福邨廣仁樓地下19號

開放時間:

星期一至五: 8:30am - 1pm ; 2:30pm - 7pm

星期六: 8:30am - 1pm ; 2pm - 5pm

電話: 2638 3846

WhatsApp: 6226 5342

Whatsapp 回覆時段:

星期一至五: 9am - 5pm

星期六: 9am - 1pm

English translation:

Token no.:<br><br>**Token for FREE rotavirus vaccine (first dose)**<br><br>Please bring this token to the **Kwong Fuk Community Health Center**# **(appointment needed)**. Your child can receive the first dose of rotavirus vaccine* at **no cost (including vaccine cost and consultation fee)**.<br><br>The first dose needs to be received at around 6-10 weeks old (must be before 20 weeks old).<br><br>Study no.:<br>Gender:<br>Month of birth:<br>EAS Nelson<br>Principal investigator<br>Increasing rotavirus vaccine uptake: a randomised controlled trail<br># Please refer to the details of the Community Health Center at the back of the token<br>* The full course includes 2 doses	**Kwong Fuk Community Health Center (appointment needed)**<br><br>Address: 19, G/F, Kwong Yan House, Kwong Fuk Estate, Tai Po, New Territories<br>Opening hours:<br>Mon-Fri: 8:30am - 1pm; 2pm - 7pm<br>Sat: 8:30am - 1pm; 2pm - 5pm<br>Tel.: 2638 3846<br>WhatsApp: 6226 5342<br>WhatsApp responding period:<br>Mon-Fri: 9am - 5pm<br>Sat: 9am - 1pm
Token no.:<br><br>**Token for FREE rotavirus vaccine (second dose)**<br><br>Please bring this token to the **Kwong Fuk Community Health Center**# **(appointment needed)**. Your child can receive the second dose of rotavirus vaccine* at **no cost (including vaccine cost and consultation fee)**.<br><br>The second dose needs to be received at least 4 weeks after the first dose, and by 24 weeks old.<br><br>Study no.:<br>Gender:<br>Month of birth:<br>EAS Nelson<br>Principal investigator<br>Increasing rotavirus vaccine uptake: a randomised controlled trail<br># Please refer to the details of the Community Health Center at the back of the token<br>* The full course includes 2 doses	**Kwong Fuk Community Health Center (appointment needed)**<br><br>Address: 19, G/F, Kwong Yan House, Kwong Fuk Estate, Tai Po, New Territories<br>Opening hours:<br>Mon-Fri: 8:30am - 1pm; 2pm - 7pm<br>Sat: 8:30am - 1pm; 2pm - 5pm<br>Tel.: 2638 3846<br>WhatsApp: 6226 5342<br>WhatsApp responding period:<br>Mon-Fri: 9am - 5pm<br>Sat: 9am - 1pm
