# Supplementary material for: Multiple-component interventions to increase rotavirus vaccine uptake in children: a randomised controlled trial
Source: Lancet Reg Health West Pac. 2024 Aug 5;50:101153. doi: 10.1016/j.lanwpc.2024.101153 (PMC11357879; doi:10.1016/j.lanwpc.2024.101153)
Supplement: IRVU Protocol NTEC [file mmc2.pdf]

Increasing rotavirus vaccine uptake: a randomised controlled trial

Study Protocol

1. General information

**Protocol Number:**	IRVU-001: 20200310
**Protocol Title:**	Increasing rotavirus vaccine uptake: a randomised controlled trial
**Version number with Date:**	Version 3.1 dated 25 Jan 2023
**Study Monitor:**	Department of Paediatrics, The Chinese University of Hong Kong, Prince of Wales Hospital, Shatin, Hong Kong<br>Tel: +852 35052861, Fax: +852 26360020
**Person authorised to sign protocol:**	Prof NELSON, Edmund Anthony Severn
**Investigators:**	Prof NELSON, Edmund Anthony Severn<br>Department of Paediatrics, The Chinese University of Hong Kong<br>+852 35052861, tony-nelson@cuhk.edu.hk<br><br>Dr YEUNG, Hoi Ting Karene<br>Department of Paediatrics, The Chinese University of Hong Kong<br>+852 35052917, karene@link.cuhk.edu.hk<br><br>Prof TAM, Wing Hung<br>Department of Obstetrics and Gynaecology,<br>The Chinese University of Hong Kong<br>+852 35052802, tamwh@cuhk.edu.hk<br><br>Dr FUNG, Po Gee Genevieve<br>Department of Paediatrics, The Chinese University of Hong Kong<br>+852 9469 8094, fungpg@hotmail.com<br><br>Dr LIU King Chun<br>Department of Paediatrics and Adolescent Medicine,<br>United Christian Hospital<br>+852 3949 4000, lks778@ha.org.hk
**Project site:**	Department of Paediatrics, The Chinese University of Hong Kong, Prince of Wales Hospital, Shatin, Hong Kong<br><br>Department of Paediatrics and Adolescent Medcine, United Christian Hospital, Kwun Tong, Hong Kong

**Compliance:**	This study will be conducted in compliance with Declaration of Helsinki.

2. Background information

Rotavirus is a major cause of morbidity and mortality globally, both in developed and developing countries. There were an estimated 128,500 rotavirus deaths globally in 2016 (1). Almost every child will be infected with rotavirus by the age of 5 years irrespective of where they live, and 35%-40% of diarrheal hospitalizations in this age group are due to rotavirus (2). Besides diarrhoea, rotavirus gastroenteritis is associated with systemic infection, seizures, cognitive deficits, growth faltering, and economic impacts on families. Although it is well recognised that improvements in sanitation and safe water reduce the risk of bacterial and parasitic enteric infections, it is much less understood that clean and safe-water environments are not strongly protective against rotavirus infections. Vaccination is the principal preventive strategy against severe rotavirus infections and the World Health Organization (WHO) recommends that rotavirus vaccine be introduced into all national immunisation programmes (3).

Children in Hong Kong are provided with free vaccinations against 12 antigens in Maternal and Child Health Centres (MCHC) and at school (Primary 1, 5 and 6) under the Hong Kong Government's Childhood Immunisation Programme (CIP). Vaccines not included in the routine CIP schedule, such as rotavirus vaccine, influenza vaccine, and Haemophilus influenzae type b vaccine, can be obtained if parents take their children to doctors working in the private sector. Uptake of rotavirus vaccine was found to be 33.3% in an immunisation survey in preschool children aged 2-5 years conducted by the Department of Health in 2015 (unpublished information obtained from personal communication and from a published study (4)). This uptake rate is much lower that than the high immunisation coverage rates of vaccines included in the CIP of over 95% (4;5).

We have previously assessed the disease burden of rotavirus infection in hospitalised children in Hong Kong over 14 rotavirus seasons (1 July 1997 to 31 March 2011) (6). The unadjusted incidence rates per 100,000 person-years based on any International Classification of Disease discharge diagnosis of rotavirus (008.61) were: 249 (0-<1m); 612 (1-<2m); 1066 (2-<6m); 1383 (6-<11m); 959 (1-<2y); 406 (2-<3y); 233 (3-<4y); 124 (4-<5y). This analysis suggested that a discharge diagnosis of rotavirus alone likely under-reported true incidence by a factor of between 1.59-2.02 in children below 5 years of age. Overall we estimated that the cumulative risk of admission with rotavirus infection in Hong Kong children below 5 years was 1 in 33 children.

In 2014/15 we conducted a test-negative case-control study of rotavirus vaccine effectiveness in Hong Kong and showed that the unconditional vaccine effectiveness against hospitalisation for administration of at least one dose of either rotavirus vaccines was 92% (95% confidence interval [CI]: 75%, 98%) (7). The conditional analyses by age only and both age and admission date showed 96% (95% CI: 72%, 100%) and 89% (95% CI: 51%, 97%) protection against rotavirus hospitalisation respectively.

In a Knowledge, Attitudes and Practices study of 500 mother-infant pairs, we showed that family monthly household income had direct effects on both perceived benefits of rotavirus vaccine ($\beta = 0.16$) and self-efficacy to vaccinate the children ($\beta = 0.16$) (8). The highest income quartile were 1.5 times more likely to vaccinate their children with rotavirus vaccine.

In view of the relatively low uptake rate of this safe and effective vaccine, and the very significant disease burden in Hong Kong children, we believe that there is an important research need to assess intervention strategies that could increase rotavirus vaccine uptake in Hong Kong children, particularly in the lower socioeconomic groups. We propose to assess an intervention designed to increase rotavirus vaccine coverage in children by removing financial barriers and increasing mothers' knowledge in rotavirus infection and vaccination.

3. Hypotheses and objectives

Hypotheses:

1. The uptake of rotavirus vaccine in Hong Kong is associated with socioeconomic status and removal of financial barriers will increase uptake of the vaccine in all socio-economic groups but proportionately more in the low-income groups.
2. The uptake of rotavirus vaccine in Hong Kong is associated with parents' knowledge and attitudes in relation to rotavirus vaccine and infection and providing key information on rotavirus infection and vaccination will increase uptake of the vaccine.

Aims:

1. To determine whether removal of financial barriers can increase uptake of rotavirus vaccine in children.
2. To determine whether provision of key information on rotavirus infection and vaccination can increase uptake of rotavirus vaccine in children.

4. Study design

This is a prospective, randomised controlled trial (Active control: 2 intervention groups and 1 control group) of two intervention packages designed to increase the uptake of rotavirus vaccine in children.

Subjects

The following criteria will be used for selection of study participants: (i) postpartum mothers from the study hospitals; (ii) aged older than 18 years; (iii) Cantonese speaking and able to read traditional Chinese; (iv) have a plan to remain in Hong Kong with the infant after delivery for at least 8 months (upper age limit for giving rotavirus vaccine according to product information); (v) no obvious cognitive abnormality; (vi) no serious obstetrical complications and baby is full-term (≥37 weeks of gestation) with no congenital abnormalities; and (vii) can provide signed informed consent. Subjects who cannot meet the inclusion criteria would be excluded. A convenience sample of women who meet the inclusion criteria will be invited to participate.

As a token of appreciation, we will offer supermarket coupons with equivalent value of HKD200 (USD26) to subjects on completion of the study by registered mail, in accordance with local experience and international practice. Upon receiving the incentive, participants will be asked to sign on a form of

acknowledgement of receipt. Dates of incentive sent and received and the return of the acknowledgement of receipt will be recorded on a study log sheet as a governance system.

Interventions

Control Group

Subjects in the control group will receive the publicly available information from the Centre for Health Protection (CHP) about rotavirus infection (https://www.chp.gov.hk/en/healthtopics/content/24/38.html) at postnatal wards.

Intervention Group

There will be two intervention groups. Subjects in both intervention groups will receive the same publicly available information from the CHP about rotavirus infection as the control group. This can ensure all subjects are aware of rotavirus disease and the vaccine. In addition intervention group 1 will receive an intervention package by post one to two days after discharge from hospital, which includes: (i) key information about rotavirus infection and vaccination, and (ii) a hyperlink of a webpage showing clinics in the private sector that provide rotavirus vaccines. A text message reminder for vaccination with the same hyperlink of private clinics will be sent at the children's age of 6 to 8 weeks. Intervention group 2 will receive the same intervention as the intervention group 1, and additionally be given the contact numbers and addresses of a specific community health centre within the United Christian Nethersole Community Health Service (UCN) that will provide rotavirus vaccines free of charge to their infants and in reasonable proximity to their home. A token will be attached in the intervention package to intervention group 2. With the token presented at the health centres, subjects can receive rotavirus vaccine free of charge.

Methods

Mothers will be enrolled in the study at the postnatal wards of two public hospitals (Prince of Wales Hospital and United Christian Hospital). After consent has been obtained and questionnaires have been completed, a study Research Assistant (RA) not involved with other follow-ups will randomly allocate all participants to either control or two intervention groups using block randomisation with 1:1:1 ratio (9). Each group will have 262-263 participants. We will use statistical software R version 3.5.2 to randomly generate the intervention allocation in random block sizes of 6 to 12 with block size kept unknown to investigators and the RA carrying out interviews. This precaution maintains concealment. Once the intervention has been allocated, this RA will not be blinded to participants' group assignment, but the other RA conducting telephone interviews, investigators and participants will be blinded to participants' group allocation. Families in all groups will then be given an envelope containing the standard information about rotavirus infection. The intervention group 1 will receive additional information about rotavirus infection and vaccination and a hyperlink of a webpage showing clinics in the private sector that provide rotavirus vaccines by post one to two days after discharge from hospital and a RA will call and explain the information within a month after the package sent. Then a text message reminder at the children's age of 6 to 8 weeks will be provided. The intervention group 2 will receive the same information as the intervention group 1, and additionally be given information on how to contact a specific UCN health centre where they can receive rotavirus vaccine free of charge and in reasonable proximity to their home.

Data collection

The study data will be collected at two time-points: (i) a face-to-face interview followed by a self-administered questionnaire at enrolment in postnatal wards; and (ii) an online self-administered questionnaire at children's age of approximately 8 months. In the first questionnaire, we will collect information on the key demographic information and mother's knowledge, attitudes and practices in relation to rotavirus vaccine. In the online questionnaire, we will collect the child's rotavirus vaccination history at 8 months old and an electronic copy of the child's vaccination record card. In addition all participants will be asked about the knowledge, attitudes and practices in relation to rotavirus vaccine. Participants will be followed-up by phone if the final questionnaire is not completed.

Knowledge, attitudes and practices of mothers will be assessed by questionnaires based on Health Belief Model (HBM). The HBM is a simple, widely used framework to explain human health decision-making and behaviour (10;11). It was developed in the 1950s and improved in 1980s (12-14). It contains six concepts to predict how humans make decisions. These include perceived susceptibility and severity of illnesses, perceived benefits and barriers of interventions, cues to action and self-efficacy. The questionnaires are based on previous intervention study to increase influenza vaccine uptake (15). The modified questionnaire will be pilot-tested in approximately 30 mothers at the postnatal wards of the two study hospitals so as to ensure the internal consistency of questionnaire items within constructs is acceptable. Questionnaires will then be refined if necessary.

5. Selection of subjects

The provision of publicly available information from the CHP about rotavirus infection to all participants may increase their knowledge of the vaccine in subjects who do not know the vaccine before joining the study. To ensure we will have enough sample to examine the study objectives, we assume the baseline rotavirus vaccine uptake would be 40%. We estimate the intervention 2 will increase the subjects' rotavirus vaccine uptake from 40% to at least 55%. Anticipating 20% drop-out, based on a required power of 80% and a significance level of 0.05, by using G*Power version 3.1.9.2, the sample size required in each group is 262-263, and 788 in total. 459 and 329 subjects will be recruited in the Prince of Wales Hospital and United Christian Hospital respectively.

6. Study tools

The intervention will include: (i) standard information about rotavirus infection (same as that for the controls) (ii) key information about rotavirus infection and vaccination, (iii) a hyperlink of a webpage showing clinics in the private sector that provide rotavirus vaccines, (iv) vaccination reminders and (v) contacts of specific community health centres and a token to receive rotavirus vaccines free of charge.

7. Safety

Not applicable as this research does not involve any safety issue. Side effects of rotavirus vaccination are rare, usually mild, and may include fussiness, diarrhoea and vomiting. Some studies have shown a small

increased risk of intussusception within a week after vaccination but the World Health Organization and other health authorities advise that the benefits of rotavirus vaccination far outweigh this risk.

8. Statistics

Exposures

Type of intervention: standard information about rotavirus infection (control group and two intervention groups) plus (i) key information about rotavirus infection and vaccination, (ii) a hyperlink of a webpage showing clinics in the private sector that provide rotavirus vaccines, (iii) vaccination reminders and (iv) contacts of specific community health centres and a token to receive rotavirus vaccines free of charge (intervention group 2 only).

Outcome measures

Primary Outcome measure
Uptake rate of rotavirus vaccine in children at 8 months.

Secondary Outcome measure
1. Knowledge, attitudes and practices of mothers in relation to rotavirus vaccine before and after receiving control or intervention information.
2. Uptake rate of rotavirus vaccine in children at 8 months by family socioeconomic status.

Statistical analysis

To ensure data quality, the data will be double-entered and validated using EpiData. Intention-to-treat analysis will be used with missing status of rotavirus vaccination taken as no vaccination. An initial univariate analysis will be performed on all variables to derive means, standard deviations and ranges. These initial results will be examined for possible errors and corrected as necessary. To assess the usefulness of the interventions, chi-squared tests will be used to compare rotavirus vaccine uptakes between control and intervention groups. To determine whether removal of financial barriers can increase uptake of rotavirus vaccine in children (study objective 1), we will compare intervention group 2 with the intervention group 1. To determine whether provision of key information on rotavirus infection and vaccination can increase uptake of rotavirus vaccine in children (study objective 2), we will compare intervention group 1 with the control group. We will use multiple regression models to assess the association between the rotavirus vaccination uptake rate and mothers' knowledge and attitudes before and after delivering intervention materials. The models will then be compared to assess the factors influenced by the interventions. To determine the change in knowledge and attitudes after receiving intervention information, permutation tests (16) will be performed on the paired data. All statistical analysis will be performed using statistical software R version 3. 5.2 and a two-tailed p-value <0.05 will be taken as statistically significant.

9. Access to data

All personal information will be kept confidential and the use of the data collected will only be restricted in the office of the Department of Paediatrics of The Chinese University of Hong Kong. Access to data is only limited to authorised members of research team under the direction of the principal investigator.

10. Quality control and quality assurance

Face-to-face interviews will be conducted in United Christian Hospital and Prince of Wales Hospital. Telephone follow-ups and data analysis will be carried out in the Department of Paediatrics of The Chinese University of Hong Kong using their office space. Principal investigator and co-investigators will supervise the project to ensure it progresses appropriately.

11. Ethics

Written informed consent will be sought from each participant and they will be informed of the potential risks and benefits clearly before signing the informed consent from. To protect participants' privacy, all research data would be handled in line with Hospital Authority / Hospital's policy in handling / storage / destruction of patients' medical records. Research data will be locked in cabinets where the department or ward keeps patients' confidential information. Electronic data will be saved in a secured computer of the hospital or university with restricted access. The protocol complies with ICH-GCP.

12. Data handling

All data collected will be locked, held in anonymous files and analysed within the Department of Paediatrics of The Chinese University of Hong Kong.

13. Insurance

Not applicable.

14. Publication policy

Papers approved by the study team will be published in appropriate peer reviewed journals.

Reference list

(1) Troeger C, Khalil IA, Rao PC, Cao S, Blacker BF, Ahmed T, et al. Rotavirus Vaccination and the Global Burden of Rotavirus Diarrhea Among Children Younger Than 5 Years. JAMA Pediatr 2018 Oct 1;172(10):958-65.

(2) Parashar UD, Nelson EA, Kang G. Diagnosis, management, and prevention of rotavirus gastroenteritis in children. BMJ 2013 Dec 30;347:f7204.

(3) World Health Organization. Rotavirus vaccines: WHO position paper - January 2013. World Health Organization; 2013. Report No.: 5.

(4) Chan D. Immunisation Coverage for Children Aged Two to Five: Findings of the 2015 Immunisation Survey. Communicable Diseases Watch 2017;14(6):23-7.

(5) Wong A. World Immunization Week 2013. Communicable Diseases Watch 2013;10(8):29-32.

(6) Chiang GP, Nelson EA, Pang TJ, Law SK, Goggins W, Chan JY, et al. Rotavirus incidence in hospitalised Hong Kong children: 1 July 1997 to 31 March 2011. Vaccine 2014 Mar 26;32(15):1700-6.

(7) Yeung KH, Tate JE, Chan CC, Chan MC, Chan PK, Poon KH, et al. Rotavirus vaccine effectiveness in Hong Kong children. Vaccine 2016 Sep 22;34(41):4935-42.

(8) Yeung KHT. Identifying and Lowering Barriers to Optimise Interventions for Pneumonia and Diarrhoea in Children. The Chinese University of Hong Kong 2017;http://repository.lib.cuhk.edu.hk/en/item/cuhk-1839341 (Accessed 7 Nov 2019).

(9) Altman D, Bland J. How to randomise. BMJ 1999 Sep 11;319.

(10) Champion VL, Skinner CS. The Health Belief Model. In: Glanz K, Rimer BK, Viswanath K, editors. Health behavior and health education: theory, research, and practice. 4 ed. San Francisco: Jossey-Bass; 2008. p. 45-65.

(11) World Health Organization. Health education: theoretical concepts, effective strategies and core competencies. World Health Organization; 2012.

(12) Hochbaum GM. Public participation in medical screening programs; a socio-psychological study. Washington: 1958.

(13) Rosenstock IM. The Health Belief Model and Preventive Health Behavior. Health Educ Behav 1974;2(354).

(14) Rosenstock IM, Strecher VJ, Becker MH. Social learning theory and the Health Belief Model. Health Educ Q 1988;15(2):175-83.

(15) Yeung KHT, Tarrant M, Chan KCC, Tam WH, Nelson EA. Increasing influenza vaccine uptake in children: A randomised controlled trial. Vaccine 2018;36(37):5524-35.

(16) Legendre P, Legendre L. Statistical testing by permutation. Numerical ecology. 2nd Edition ed. Amsterdam: Elesevier Science BV; 1998. p. 17-26.
